# Comparison of ozone formation attribution techniques in the northeastern United States

**DOI:** 10.5194/gmd-16-2303-2023

**Published:** 2023-04-28

**Authors:** Qian Shu, Sergey L. Napelenok, William T. Hutzell, Kirk R. Baker, Barron H. Henderson, Benjamin N. Murphy, Christian Hogrefe

**Affiliations:** U.S. Environmental Protection Agency, Research Triangle Park, NC 27711, USA

## Abstract

The Integrated Source Apportionment Method (ISAM) has been revised in the Community Multiscale Air Quality (CMAQ) model. This work updates ISAM to maximize its flexibility, particularly for ozone (O_3_) modeling, by providing multiple attribution options, including products inheriting attribution fully from nitrogen oxide reactants, fully from volatile organic compound (VOC) reactants, equally from all reactants, or dynamically from NO_*x*_ or VOC reactants based on the indicator gross production ratio of hydrogen peroxide (H_2_O_2_) to nitric acid (HNO_3_). The updated ISAM has been incorporated into the most recent publicly accessible versions of CMAQ (v5.3.2 and beyond). This study’s primary objective is to document these ISAM updates and demonstrate their impacts on source apportionment results for O_3_ and its precursors. Additionally, the ISAM results are compared with the Ozone Source Apportionment Technology (OSAT) in the Comprehensive Air-quality Model with Extensions (CAMx) and the brute-force method (BF). All comparisons are performed for a 4 km horizontal grid resolution application over the northeastern US for a selected 2 d summer case study (9 and 10 August 2018). General similarities among ISAM, OSAT, and BF results add credibility to the new ISAM algorithms. However, some discrepancies in magnitude or relative proportions among tracked sources illustrate the distinct features of each approach, while others may be related to differences in model formulation of chemical and physical processes. Despite these differences, OSAT and ISAM still provide useful apportionment data by identifying the geographical and temporal contributions of O_3_ and its precursors. Both OSAT and ISAM attribute the majority of O_3_ and NO_*x*_ contributions to boundary, mobile, and biogenic sources, whereas the top three contributors to VOCs are found to be biogenic, boundary, and area sources.

## 1 Introduction

Tropospheric O_3_ is a critical air pollutant that endangers human health (WHO, 2013) and sensitive vegetation (Booker et al., 2009) and contributes to climate change (Jacob and Winner, 2009). It is produced through nonlinear photochemical reactions of carbon monoxide (CO), volatile organic compounds (VOCs), and nitrogen oxides (NO_*x*_ = NO + NO_2_) with sunlight (Atkinson, 2000). In the United States, the national average ambient O_3_ concentration has decreased by 22 % since 1990, owing to regulations such as the Clean Air Act (CAA) on NO_*x*_ and VOC emissions (Simon et al., 2015). Long-term space observations have also confirmed the improvement in air quality (Duncan et al., 2013; Lamsal et al., 2015). However, many major metropolitan areas continue to exceed the O_3_ National Ambient Air Quality Standards (NAAQS) set by the U.S. Environmental Protection Agency (U.S. EPA). To continue to reduce O_3_ levels, it is critical to develop effective emission control strategies as has been done for other pollutants (Lefohn et al., 1998; Reitze, 2004; Cooper et al., 2015). The effectiveness of any O_3_ control strategy hinges on accurately quantifying the contributions of various precursor emissions to O_3_ formation.

Numerous techniques have been used to characterize and quantify the relationship between emission sources and O_3_ concentrations, including statistical methods, model sensitivity simulations, and model source apportionment approaches, each with its own set of advantages and disadvantages (Cohan and Napelenok, 2011). While some traditional receptor-based methods based on chemical mass balance (CMB, Hidy and Friedlander, 1971), such as effective variance solution (EV; Watson et al., 1984) and positive matrix factorization (PMF, Paatero and Tapper, 1994), produce insightful results when measurements are taken at a specific receptor, they are typically applied to speciated VOC and particulate matter (PM) and are also constrained by the relative sparsity of observations in space and time, rendering them unsuitable for regional and national O_3_ precursor emission control strategies. Alternatively, three-dimensional air quality models (AQMs) allow for the quantification of O_3_ source contributions at regular intervals over longer periods and wider spatial distributions. The most basic source apportionment (SA) technique in the context of an AQM is to conduct source sensitivity simulations using the brute-force (BF) method, in which several simulations are conducted, each with one source eliminated or reduced. The differences in the output fields compared to the baseline simulation are then attributed to the eliminated or reduced source (e.g., Marmur et al., 2005). BF has some limitations when used to determine total source culpability of O_3_ due to the pollutants’ nonlinear dependence on both relative and absolute VOC and NO_*x*_ concentrations. For example, removing NO_*x*_ may lead to an increase in O_3_ concentrations in the vicinity of large NO emissions (e.g., power plants), as a result of net conversion of O_3_ to NO_2_ (Gillani and Pleim, 1996), or at nighttime when NO_*x*_ titration cannot be balanced by the photolysis of NO_2_. In some cases, where a source contributes a substantial portion of total NO_*x*_ or VOC emissions, complete source removal for the purposes of source apportionment calculation may also substantially alter the underlying chemical regime for formation of secondary pollutants such as O_3_. Further, to separate the contributions and interactions of “*n*” sources, Stein and Alpert (1993) showed that BF would require 2 to the power of the number of sources of simulations (2^*n*^). This is quickly impractical, leading to a subset of BF simulations with unknown interactions. As a result, summarizing the O_3_ change in response to multiple brute-force emission source simulations can make it difficult to interpret the cumulative effect of those emissions on O_3_ (Kwok et al., 2015).

Reactive tracer or tagged species SA methods for O_3_ have also been incorporated in AQMs. These tracers are usually additional species added to the AQM to track the contributions of pollutants from specific source categories. They undergo the same atmospheric processes as the bulk chemical species within the model (Kwok et al., 2015). As one example, Ozone Source Apportionment Technology (OSAT) within the Comprehensive Air-quality Model with Extensions (CAMx) quantifies the contributions of various emission sectors, source regions, and initial and lateral boundary conditions to simulated O_3_ concentrations (Ramboll Environ, 2015). OSAT allocates instantaneous O_3_ formation to either NO_*x*_ or VOCs based on the ratio of hydrogen peroxide (H_2_O_2_) to nitric acid (HNO_3_) production (Dunker et al., 2002). O_3_ formation is classified as being NO_*x*_-limited or VOC-limited formation based on the gross production of H_2_O_2_ (PH_2_O_2_) and HNO_3_ (PHNO_3_). When the ratio (PH_2_O_2_/PHNO_3_) is above 0.35, the formation is classified as NO_*x*_-limited and VOC-limited otherwise (Sillman, 1995). If the photochemical formation of O_3_ (PO_3_) occurs in a NO_*x*_-limited regime, the NO_*x*_ tracers are used to attribute PO_3_ proportionally to the emissions sources that contributed to the NO_*x*_ concentrations. Otherwise, VOC tracers are used to attribute PO_3_ to the sources that contributed to the VOC concentrations (Dunker et al., 2002; Kwok et al., 2015). The OSAT formulation was recently changed (OSAT3) to track all forms of NO_*x*_ to account for NO_*x*_ recycling, which occurs when NO_*x*_ is converted to another form of NO_*x*_ (e.g., peroxyacetyl nitrate (PAN) or HNO_3_) and then converted back to NO_*x*_. OSAT has been used to support policy assessments (e.g., U.S. EPA, state government agencies; Ramboll Environ, 2015, 2022) and for scientific research purposes (Li et al., 2012; Zhang et al., 2017; Shu et al., 2020).

Additionally, the Integrated Source Apportionment Method (ISAM) within Community Multiscale Air Quality (CMAQ) has shown promising results for O_3_ tagging (Kwok et al., 2015). Recent ISAM experiments have quantified the contribution of O_3_ sources to air pollution in several major cities throughout the United States and Europe (Kwok et al., 2015; Valverde et al., 2016; Karamchandani et al., 2017; Butler et al., 2018; Pay et al., 2019). The attribution of O_3_ and precursors from specific sources estimated by ISAM implemented in version 5.0 of CMAQ compared well with source-specific aircraft transect measurements (Baker et al., 2016). The ISAM algorithms have also been updated several times following the original implementation in CMAQv5.0.2.

ISAM updates presented in this study substantially increase the flexibility to the user of the CMAQ source apportionment model. These updates were intended to provide long-term flexibility within the model to accommodate newer chemical mechanisms and changed the attribution approach as detailed in Sect. 3. These flexibilities allow for apportionment of more species and allow for more methods of apportionment. Further in the paper we apply the changes to CMAQ-ISAM for a northeastern US O_3_ air quality episode and compare the results to CMAQ-BF and CAMx-OSAT. The paper is organized as follows: Sect. 2 documents the ISAM updates in detail; Sect. 3 describes the methodology for this study, which includes the base modeling configurations, simulation designs for source apportionment, tracked species classes, evaluation methods, and case study development; Sect. 4 presents the findings, including model evaluation results and comparisons of source apportionment for several species; Sect. 5 documents the running speed comparisons between CMAQ-ISAM, CAMx-OSAT, and CMAQ-BF; and the findings and their implications for future research are discussed in Sect. 6.

## 2 Source apportionment methods

### 2.1 Updates in ISAM

The ISAM implementation in the version 5.0 release of CMAQ was based on Kwok et al. (2013 and 2015). That approach was then updated starting from CMAQ version 5.3 to an attribution based on integrated reaction rates and product yields (U.S. EPA, 2019). The later versions (v5.3.2 and beyond) of CMAQ-ISAM (U.S. EPA, 2022a) employ an apportionment scheme that assigns products of each chemical reaction to sources based on reactant stoichiometry. For example, the isoprene peroxy radical (ISO_2_) reacts with nitric oxide (NO) to produce several different stable and radical species as represented in the CB6R3 chemical mechanism by the following Reaction (R1).


(R1) $${\text{ISO}}_{2}+\text{NO}=0.1\times \text{INTR}+0.9\times {\text{NO}}_{2}+0.673\times \text{FORM}+0.9\times \text{ISPD}+0.818\times {\text{HO}}_{2}+0.082\times {\text{XO}}_{2}\text{H}+0.082\times {\text{RO}}_{2}.$$

In addition to nitrogen dioxide (NO_2_), the products include isoprene nitrate (INTR), formaldehyde (FORM), hydroperoxy radicals (HO_2_), alkoxy radicals (XO_2_H), peroxy radicals (RO_2_), and other isoprene reaction products (ISPD). ISO_2_ is a product of the oxidation of isoprene, which originates from overwhelmingly biogenic sources. NO is typically emitted from anthropogenic combustion processes, with a much smaller natural component originating from lightning strikes and microbial soil processes on the global scale (Jacquemin and Noilhan, 1990; Yienger and Levy, 1995). Thus, the reactants are approximately half from biogenic sources and half from anthropogenic sources, so the reaction’s products have the same attribution distribution. However, source attribution approaches, both receptor-based approaches (such as PMF) and source-based approaches (such as ISAM), are often used to understand how originally emitted NO_*x*_ and VOC from particular sources ultimately contribute to model-predicted O_3_ production. The loss of source identity through processes such as the NO_*x*_ cycle and the role of organic peroxy radicals from sources not controlling O_3_ production make it difficult to determine the culpability of emission sources. In the preceding example, the NO_2_ produced by Reaction (R1) is assigned a source that is approximately 50 % biogenic and 50 % anthropogenic. These source assignments propagate quickly when catalytic processes cause NO_2_ to cycle back to NO through photooxidation and radical oxidation. Because NO_*x*_ cycling is fast in regional air pollution models, anthropogenically emitted nitrogen species can be assigned to biogenic (or other nearby) sources downwind, so the original source identity was not retained. Reaction (R1) is just one example that illustrates the complex relationship between precursors and subsequent source identities of secondary pollutants. Many such reactions exist in modern chemical mechanisms. Some source apportionment applications, such as O_3_ source attribution assessments, focus on how sources induce O_3_ production above background levels. Nitrogen molecules should then retain their original source signatures. This approach is used by other apportionment models such as OSAT, earlier ISAM implementations (Kwok et al., 2015), and other tagging methods (Butler et al., 2018; Grewe et al., 2010).

Because attribution objectives may vary based on scale (e.g., global compared to urban) or purpose (e.g., policy or tracing chemical reactions), ISAM has been enhanced to provide additional configuration options for the user to define how secondarily formed gaseous species are assigned to sources of parent reactants (Table 1) (U.S. EPA 2022b). The existing scheme based on stoichiometrically proportional product attribution introduced in CMAQ version 5.3.2 has been retained as ISAM option 1 (ISAM-OP1). Four new options have been added so the user can configure their simulation based on the application’s goal. Each option allows for greater retention of source identity based on subsets of species in the chemical mechanism. ISAM-OP2 apportions products according to the source identity of reactive nitrogen species, including NO, NO_2_, nitrate radical (NO_3_), nitrous acid (HONO), HNO_3_, dinitrogen pentoxide (N_2_O_5_), and aerosol nitrate (ANO_3_). For example, CB6R3 contains the following reaction between the methyl peroxy radical (MEO_2_) and NO:

(R2) $${\text{MEO}}_{2}+\text{NO}=\text{FORM}+{\text{HO}}_{2}+{\text{NO}}_{2}.$$

In the original ISAM-OP1 configuration, the products of Reaction (R2), FORM, HO_2_, and NO_2_ inherit source identities proportional to the source identities of the reactants (MEO_2_ and NO). However, ISAM-OP2 apportions the product to be from the source identity of NO (presumed predominantly anthropogenic) because NO is a weighted nitrogen-containing species. When a reaction’s reactants do not include any of the weighted species, products are apportioned to source identities using the same methodology used in OP1.

ISAM-OP3 expands OP2’s list of weighted species to include VOC species identified as important to O_3_ production. In CB6R3, this includes aldehydes (ALD_2_ and ALDX), FORM, acetone (ACET), lumped ketones (KET), peroxy operators (XO_2_ and XO_2_H), ISO_2_, acetyl peroxy radicals (C_2_O_3_ and CXO_3_). Therefore, products of reactions containing these VOCs in addition to the nitrogen species of OP2 as reactants would inherit these species’ source identities. For example, ALD_2_ reacts with the NO_3_ as follows in CB6R3.


(R3) $${\text{ALD}}_{2}+{\text{NO}}_{3}={\text{C}}_{2}{\text{O}}_{3}+{\text{HNO}}_{3}$$

The reaction’s products, C_2_O_3_ and HNO_3_, inherit identities equally divided between the sources of the reactants because ALD_2_ and NO_3_ are on the list of OP3 species. Reactions without any of these species in the reactants list, like OP2, have their products apportioned to source using OP1’s methodology when the reactants are not among the weighted ones.

ISAM-OP4 lists only VOC species and daughter products instrumental in O_3_ chemistry as defined in OP3. In the R1 example, the products are apportioned to the source identity of ISO_2_ because the other reactant, NO, is not on the list of weighted species. Similarly, the products of Reaction (R3) are attributed to the source identity of ALD_2_. As in options 2 and 3, reactions (such as Reaction R2) without any listed species are attributed as in OP1’s method.

Finally, ISAM-OP5 was added to account for the instantaneously calculated O_3_ formation regime or limiting case. The regime is determined using the ratio of PH_2_O_2_/PHNO_3_. The transition point between regimes has a default value equal to 0.35 (Sillman, 1995). For the NO_*x*_-limited regime (PH_2_O_2_/PHNO_3_ > 0.35), source identity is passed from the nitrogen species of OP2, while for the VOC-limited regime (PH_2_O_2_/PHNO_3_ ≤ 0.35) source identity is passed from the organics of OP4. These CMAQ-ISAM options, including the regime threshold value (or transition point), are accessible at runtime through the standard model run script.

### 2.2 OSAT description

The source apportionment approach implemented in CAMx is briefly recapped here. Detailed updates of all OSAT versions can be found in the CAMx official user guide (https://camx.com/Files/CAMxUsersGuide_v7.10.pdf, last access: 1 February 2023). All available versions of OSAT (including OSAT3) in CAMx separately solve for production and destruction of O_3_ with production being attributed to either NO_*x*_ or VOC emissions, depending on which is estimated to be limiting O_3_ production. When the ratio of PH_2_O_2_/PHNO_3_ exceeds 0.35, the produced O_3_ is attributed to NO_*x*_ emissions, and VOC emissions below that threshold. The CAMx source apportionment implementation includes an option (OSAT-APCA) that allows for a redirection of attribution to anthropogenic emissions in situations where the limiting precursor is biogenic. In CAMx-OSAT, O_3_ attributed to NO_*x*_ and VOCs is tracked as separate tracer groups. O_3_ tracers are first adjusted to account for O_3_ destruction processes and subsequently for net O_3_ production, which is defined as the difference between O_3_ production and O_3_ destruction based on a subset of photochemical reactions that result in O_3_ destruction. In situations where the net O_3_ production is negative (destruction reactions dominate), all the O_3_ tracers are proportionally decreased. When net O_3_ production is positive, production is assigned proportionally to the sources of those emissions (NO_*x*_ and VOC precursor tracers) at the time and place where O_3_ was made. OSAT includes a group of tracers that track odd oxygen that is consumed when O_3_ reacts with NO to form NO_2_ that can quickly photolyze and reform O_3_ through a reaction with oxygen. In this situation, the O_3_ removed from the O_3_ tracers due to the NO + O_3_ reaction is moved to the odd-oxygen tracers (which have separate NO_*x*_ and VOC tracer groups). When NO_2_ is photolyzed and O_3_ formed, a proportional amount of O_3_ is taken from the odd-oxygen tracers and moved to the O_3_ tracers.

## 3 Method

### 3.1 Base model configurations

Two models, CMAQ version 5.3.2 with modified ISAM and CAMx version 7.10 with OSAT3, are used to simulate a 1-month period during the summer of 2018 (29 July to 30 August). The summary of the two model configurations is presented in Table 2. Both models are applied to the same horizontal modeling domain with 4 km × 4 km resolution encompassing the northeastern US. This domain is nested within a larger 12 km domain that encompasses the entire contiguous United States, which is used for providing simulation boundary and initial conditions (BCs and ICs) for the 4 km domain. BCs were generated for the 12 km simulations using a hemispheric application of the GEOS-Chem model (Henderson et al., 2014) that was run for 2018. Identical ICs and BCs were applied to the two models. Anthropogenic emissions were based on version 1 of the 2016 National Emission Inventory (NEI, U.S. EPA, 2021). Electrical generating unit emissions were based on continuous emissions monitoring data from 2018 where available. On-road emissions were projected to 2018 to reflect decreases in emissions due to vehicle fleet turnover and the implementation of emission control technology in 2017. The Biogenic Emission Inventory System (Bash et al., 2016) was used to generate biogenic volatile organic compound emissions, and offline meteorology was created using the Weather Research and Forecasting (WRF, Skamarock et al., 2008) model version 3.8. CMAQ was configured using Carbon Bond 6 version 3 (CB6R3, Emery et al., 2015) for chemistry. Similarly, all base meteorological and emissions inputs for CAMx were identical to those for CMAQ but were processed using CAMx-appropriate data pre-processors (https://www.camx.com, last access: 10 March 2021). The CAMx model was configured with Carbon Bond 6 version 4 (CB6R4, Emery et al., 2016a) chemical mechanism. It is noteworthy that the major updates for CB6R4 from CB6R3 are to (i) replace full marine halogen chemistry with a condensed iodine mechanism called “I-16”, which could reduce O_3_ depletion over marine areas, and (ii) add dimethyl sulfide (DMS) chemistry. Emery et al. (2016b) demonstrated that the difference in O_3_ decrements between full halogen chemistry and I-16 is small and can be neglected over land.

### 3.2 Source apportionment simulation designs

As discussed in Sect. 2, ISAM has been updated to include a user option with five possible configurations for source apportionment approach. Here, we conduct CMAQ source apportionment simulations for all these options: ISAM-OP1, ISAM-OP2, ISAM-OP3, ISAM-OP4, and ISAM-OP5, hereafter referred to as OP1, OP2, OP3, OP4, and OP5, respectively. The OSAT3 approach was also used in the CAMx v7.10 base model for comparison with the five ISAM simulations. Hereafter OSAT3 is referred to as OSAT. A brute-force method (zeroing out the entire emission stream for tracked sources in CMAQ, hereafter referred to as CMAQ-BF) was also used to compare with the ISAM options and OSAT. A total of 11 different emission source categories were tracked using each apportionment technique. The source categories comprise four point source categories, including electricity generating units (EGU), non-electricity generating units (NONEGU), fires (FIRE), and commercial marine vessels (CMVs), and six area-source categories, including on-road mobile (ONROAD), non-road mobile (NONROAD), biogenic (BIO), railway (RAIL), airports (AIRP), and other sources (AREA). Additionally, OILGAS was tracked as a mixed category (both point and area) of emissions from the oil and natural gas industry in the domain. Total emissions from the above sectors have been displayed in Table 3. Finally, three predefined tracers for lateral boundary conditions (BCON), initial conditions (ICON), and other sources (OTHR) were also tracked for O_3_ and its precursors. OTHR is used for all remaining untagged emission categories. For example, when there are a total of 10 emission streams but only 5 of them are tracked in ISAM, the remaining 5 emission streams will be defined as OTHR. In this study, all emissions sectors were tracked as previously mentioned above for OSAT and ISAM. For CMAQ-BF, a unique CMAQ simulation for each emission source category listed above was performed by fully removing the category’s entire emission stream. CMAQ-BF apportionment was then calculated by subtracting the resulting pollutant fields from a base model simulation. However, for ICON and BCON, each was reduced by 50 % and the output field difference with the base model was scaled up by a factor of 2 to avoid numerical issues associated with very low model ICON and BCON values. As for OTHR, there is no suitable way to retain an appropriate chemical state of the troposphere after subtracting necessary emission categories, initial and boundary conditions from an original CMAQ simulation. Thus, OTHR is not being compared among CMAQ-BF, ISAM, and OSAT in this study.

### 3.3 Tracked species classes

O_3_, NO_*x*_, and VOC species were tracked by each method. As mentioned above, ISAM tracks individual oxidized nitrogen and VOC species based on selected chemical mechanisms in CMAQ, whereas OSAT tracks tracer families for each. To facilitate the comparison between the two models, the ISAM species were aggregated in the same fashion as OSAT (Table 4). However, some differences still exist since species representations between the two models are not completely the same. The nitrogen groupings NO_*y*_ and RNO_*x*_ (Table 4) were added to better elucidate the behavior of each model under different O_3_-producing chemical regimes.

### 3.4 Evaluation method and case study development

Although identical emissions and meteorological inputs are used for CAMx and CMAQ (Table 2), potential differences still exist in multiple scales and processes. Shu et al. (2017, 2022) have reported that deposition is one of the largest uncertainties between the two models when other processes are constrained. For inter-comparing ISAM and OSAT, it is not feasible to constrain all process uncertainties. Thus, we established criteria to choose representative days for ISAM and OSAT comparisons based on the performance of their parent models rather than comparing them throughout the entire simulation period to reduce the difference that may be brought on from their parent models. We initially set the correlation relationship (*R*^2^) criteria of maximum daily 8 h averaged (MDA8) O_3_ between CMAQ and CAMx to be above 0.7 to ensure that the performance of the two parent models is comparable. Next, MDA8 O_3_ was also used as the indicator for case study selection since ISAM and OSAT are normally used as regulatory application with this metric. We assess the mean bias (MB) of MDA8 O_3_ for every day to choose the days on which both models have the lowest MB for predicted MDA8 O_3_. Therefore, CMAQ- and CAMx-simulated ambient concentrations were paired in space and time with observed data from the Air Quality System (AQS, https://www.epa.gov/aqs, last access: 9 June 2021) monitoring network. Hourly concentrations of total O_3_, NO and NO_2_ were also compared to the AQS observations, and their bias statistical metrics were calculated as well.

## 4 Results

### 4.1 Model performance evaluation and case study selection

**Figure 1.**
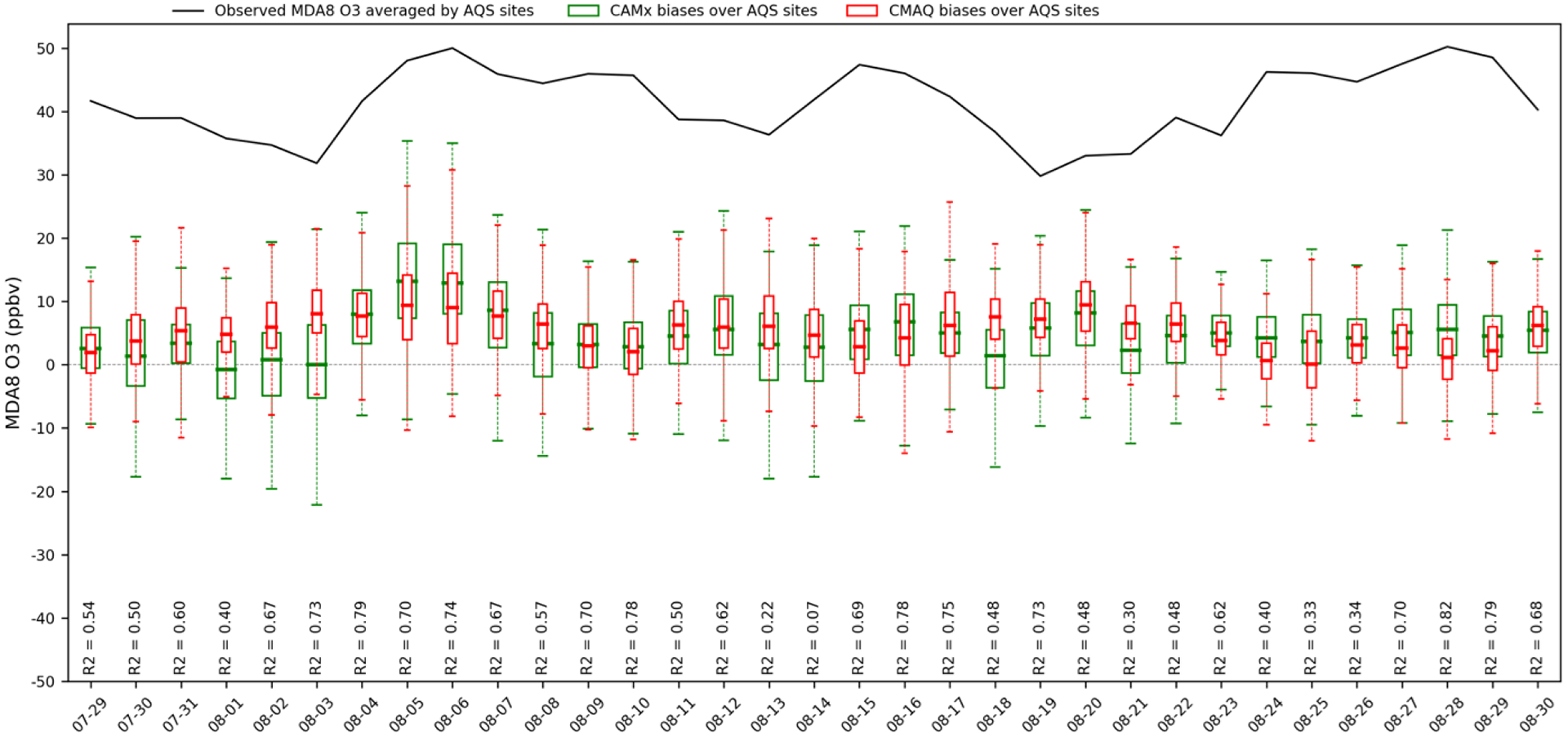
Observed site-averaged MDA8 O_3_ and its corresponding biases predicted by CMAQ and CAMx over paired AQS sites for the entire episode. *R*2 shows the correlation relationship between CMAQ and CAMx.

**Figure 2.**
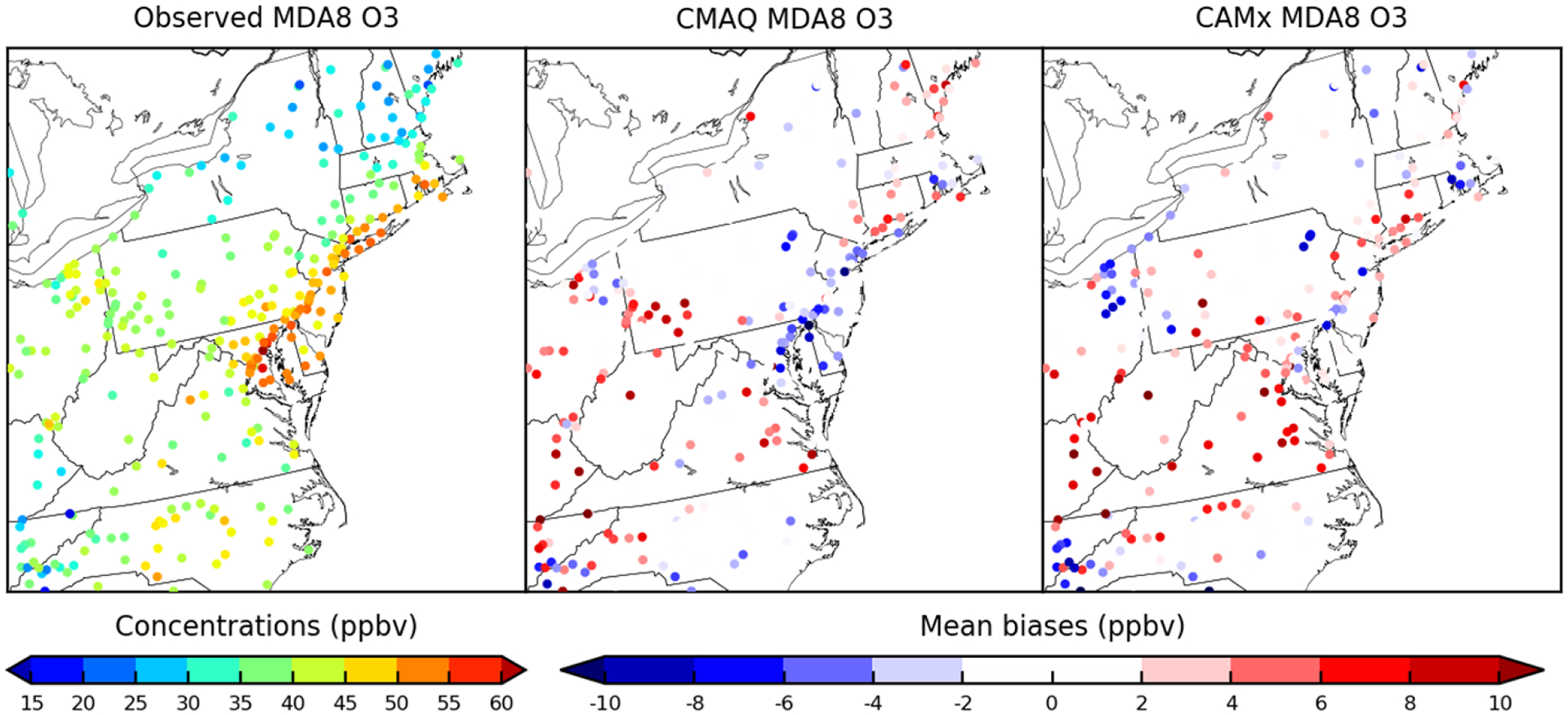
The 2 d averaged observed MDA8 O_3_ over paired sites for the northeastern US domain and its corresponding mean biases predicted by CMAQ and CAMx for the selected case study.

Figure 1 shows observed site averaged MDA8 O_3_ and its corresponding biases predicted by CMAQ and CAMx over paired AQS sites for the entire episode. Observed site averaged MDA8 O_3_ ranges from 30 to 50 ppbv. The performance of two models for predicting MDA8 O_3_ varies by paired day and monitor site with the range of biases from −23 to 35 ppbv, approximately. Table S1 in the Supplement summarizes *R*^2^ and MB of MDA8 O_3_ for each day for both models. Based on our criteria introduced in Sect. 3.4, there are 13 d on which the two models show very good correlation relationships. Among these days, two models both show good performance on predicting MDA8 O_3_ with closest MB on 9 August (CMAQ/CAMx = 3.09/2.99 ppbv) and 10 August (CMAQ/CAMx: 2.42/2.61 ppbv). For other days, either two models both have higher MB (> 10 ppbv), or their predictions do not agree well with each other, with a difference of MBs up to 8 ppbv. Therefore, 9 and 10 August were selected as a 2 d case study for source apportionment comparisons. Additional evaluations of hourly O_3_, NO and NO_2_ is available in Fig. S1 in the Supplement. From Fig. 2, MDA8 O_3_ is relatively higher over east coastal urban areas with generally over 50 ppbv but reduces to 35 ppbv at other rural areas of northeastern US domain. The two models predicted MDA8 O_3_ show very good agreement spatially, underestimating MDA8 O_3_ at sites where observed MDA8 O_3_ is high but overestimating MDA8 O_3_ at sites where O_3_ is low. Similar spatial plots of hourly paired O_3_, NO and NO_2_ can be found in the Supplement (Fig. S2). Tables 5 and 6, respectively summarize statistical metrics for MDA8 O_3_, hourly O_3_, NO and NO_2_ at all paired monitoring sites for the monthly O_3_ episode and the selected 2 d case study episode.

The metrics in Tables 5 and 6 both show consistent results with Fig. 1 as discussed above. The changes of NO and NO_2_ metrics are marginal from the monthly episode to the 2 d case. As in Fig. S1, NO and NO_2_ concentrations are less variable than O_3_ across days in the monthly episode, as a result, the comparison of NO and NO_2_ are less dependent on which day is selected. Unlike NO and NO_2_, CAMx and CMAQ performance is statistically better in the 2 d case study with lower MB for hourly O_3_ (CMAQ/CAMx = 4.67/7.02 ppbv) and MDA8 O_3_ (CMAQ/CAMx = 2.75/2.80) than the monthly episode (hourly O_3_: CMAQ/CAMx = 6.49/7.99 ppbv; MDA8 O_3_: CMAQ/CAMx = 5.30/4.18). The differences of MB, NMB and *R*^2^ between the two models also diminish for MDA8 O_3_ but increase for hourly O_3_ from the monthly episode to the 2 d episode. The statistical metrics of hourly O_3_ and MDA8 O_3_ demonstrate that the selected 2 d case is suitable for a source apportionment comparison in which CAMx and CMAQ not only both have the least-biased predictions compared to observations but also show a good agreement with each other.

### 4.2 Comparison of model source apportionment

#### 4.2.1 Temporal variations of sector contributions

**Figure 3.**
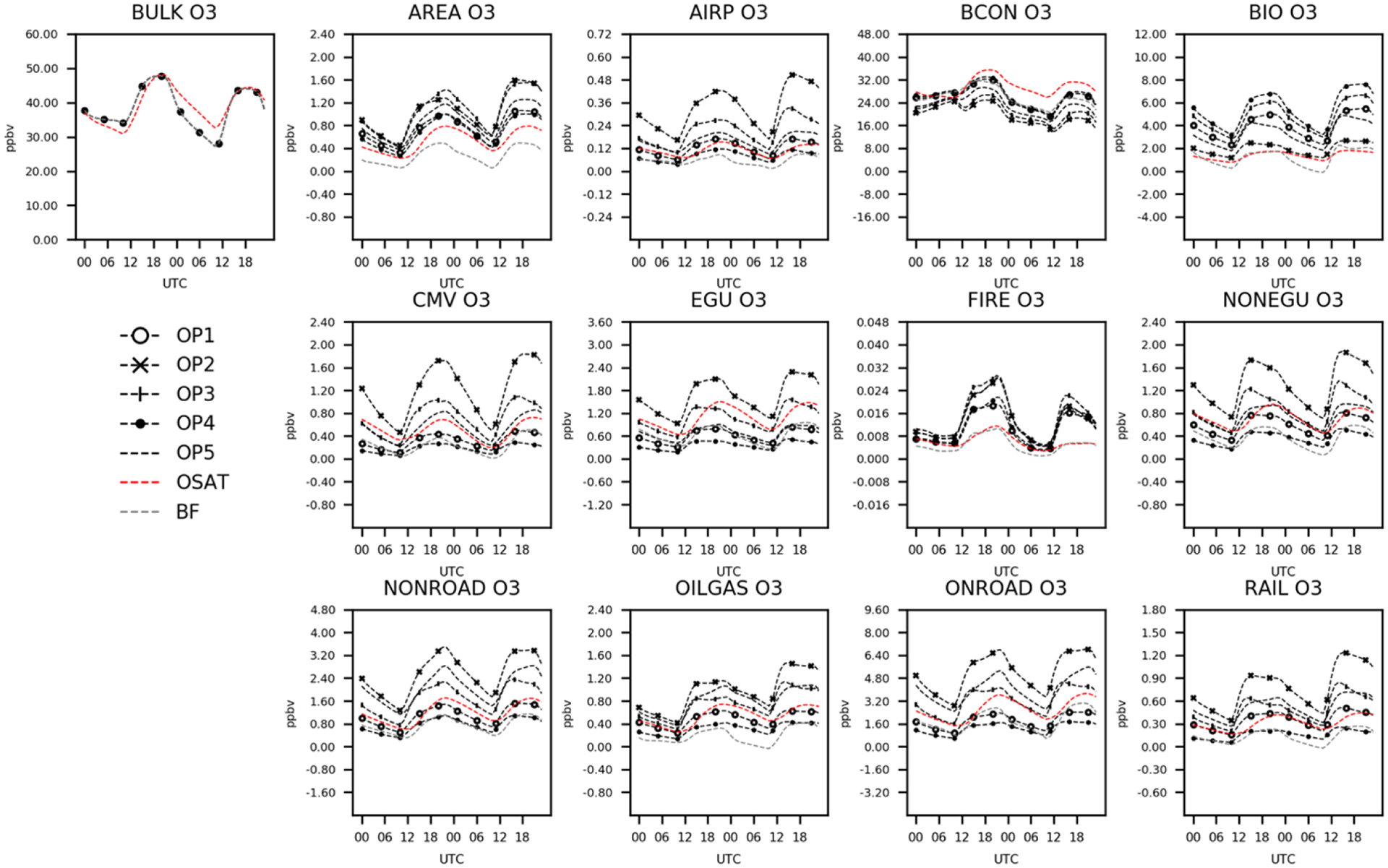
Total and attributed O_3_ concentrations to various sectors as a function of hour of day and apportionment technique.

To better understand how the ISAM model apportionment approach simulated source contributions at each time step, time series comparisons for each source were examined for O_3_ and its precursors, RNO_*x*_, and VOC for the 2 d case study. Figure 3 shows hourly variations of domain averaged predicted total O_3_ (bulk) concentrations and sector contributions for seven source apportionment simulations (OSAT, BF, ISAM OP1 to OP5). In Fig. 3, CMAQ and CAMx predict similar O_3_ concentrations during the day, but differences appear at night, with a maximum difference of 5 ppb. This disparity was discussed in Sect. 4.1 and can be mitigated by employing the MDA8 O_3_ metric. The seven source apportionment simulations yield similar diurnal trends via the trajectory of the total concentrations, but they apportion concentrations to each sector somewhat differently. Comparisons of five ISAM options reveals significant variability. OP1, which apportions uniformly according to stoichiometry, shows similar trends of apportionments for each sector as OP4, an option that always allocates products to sources with reactive VOCs and their radicals. They both apportion more BCON and BIO O_3_ but fewer contributions from all other sectors than the other three ISAM options (OP2, OP3, and OP5). Results of OP1 and OP4 would likely overestimate sensitivity to emissions to these reactants because VOCs are often available in excess. OP2 always allocates products to sources with nitrogen reactants, which prevents the attribution of NO_*x*_ to non-nitrogen reactants. Typically, these non-nitrogen reactants are common in transported (e.g., BCON) or natural sources (e.g., isoprene in BIO). As a result, OP2 decreases BCON and BIO contributions while increasing contributions from other sectors relative to OP1 and OP4.

OP5 assigns products to either reactive VOCs or NO_*x*_ based on the ratio of PH_2_O_2_/PHNO_3_, placing O_3_ contribution results for all sectors between the previous four ISAM options. OSAT, which utilizes a similar methodology to OP5, shows consistent diurnal patterns of domain averaged total O_3_ and sector contributions compared to the ISAM options but with varying magnitudes. OSAT has the largest BCON O_3_ but the lowest contributions from AREA, BIO, and FIRE. The rest of the OSAT sector contributions are between the ISAM options. Consistent with earlier findings, CMAQ-BF estimates systematically smaller O_3_ contributions for all sectors besides EGU and BCON (Kwok et al., 2015). While ISAM and OSAT appear to retain bulk mass as intended, CMAQ-BF shifts the chemical system into a different nonlinear O_3_ response to source change.

**Figure 4.**
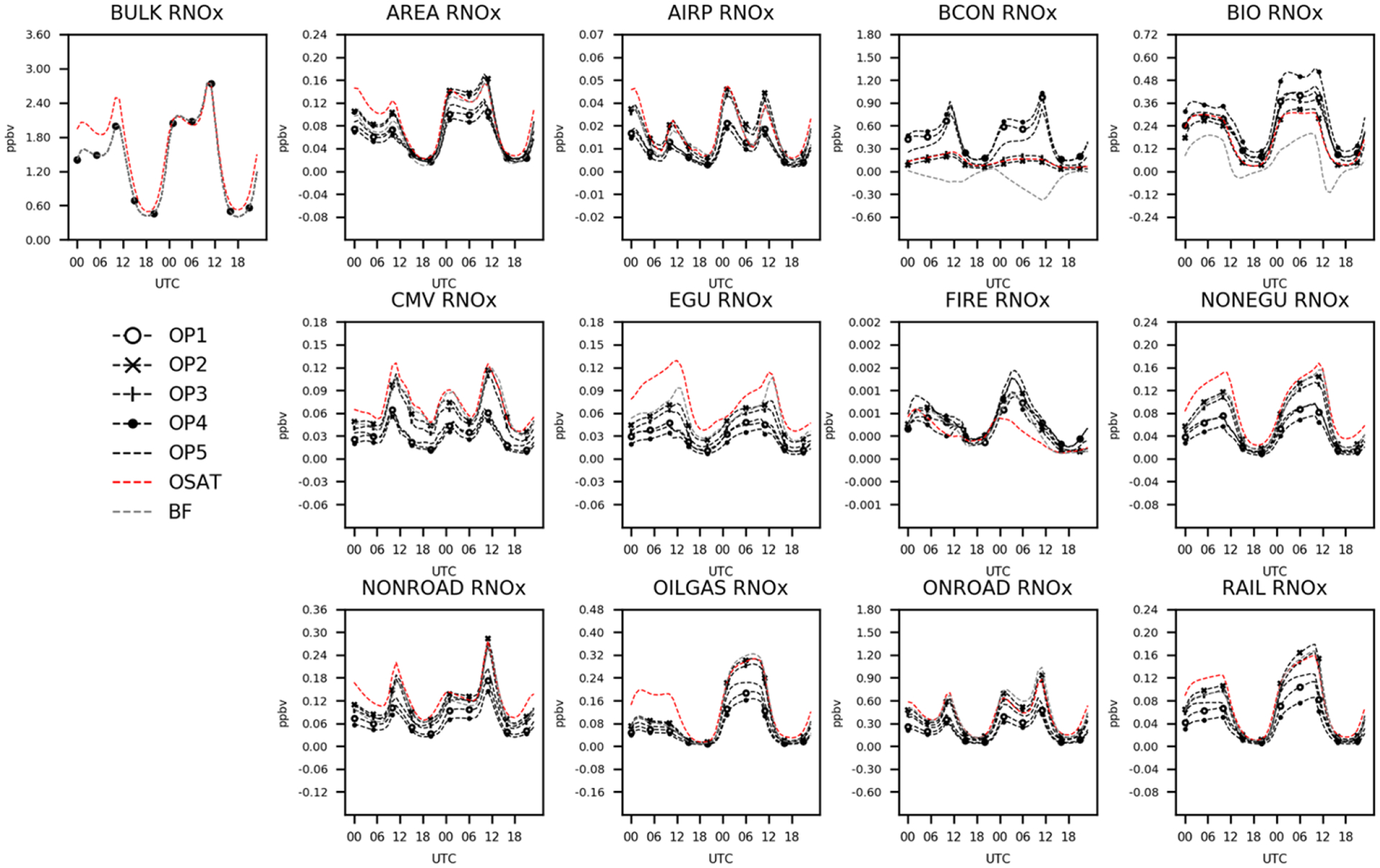
Total and attributed RNO_*x*_ concentrations to various sectors as a function of hour of day and apportionment technique.

In Fig. 4, CAMx and CMAQ predict comparable total RNO_*x*_ except for the first 12 h of the 2 d example, when OSAT values deviate from those of the other six simulations. As the total concentrations of the two models converge, OSAT exhibits similar patterns to OP2 and OP3. OP1, OP4, and OP5 show comparable results, with increased BCON and BIO RNO_*x*_ but decreased contributions from other sectors. CMAQ-BF show comparable results with OSAT, OP2, and OP3 except for BCON and BIO, which are negative for CMAQ-BF, suggesting that removing these source sectors results in a slight rise in RNO_*x*_. In previous source sensitivity and allocation investigations, it has been shown that BF may have limits when the model response contains an indirect effect coming from the influence of substances other than the direct precursors (Kwok et al., 2015; Burr and Zhang, 2011; Koo et al., 2009; Jiménez, 2004; Zhang et al., 2009). This would be particularly true in situations where emissions are a large percentage of total NO_*x*_ or VOC in a particular area. The nonlinear impacts on gas-phase chemistry realized in a source sensitivity model simulation would not be a relevant representation of culpability from that same source group.

**Figure 5.**
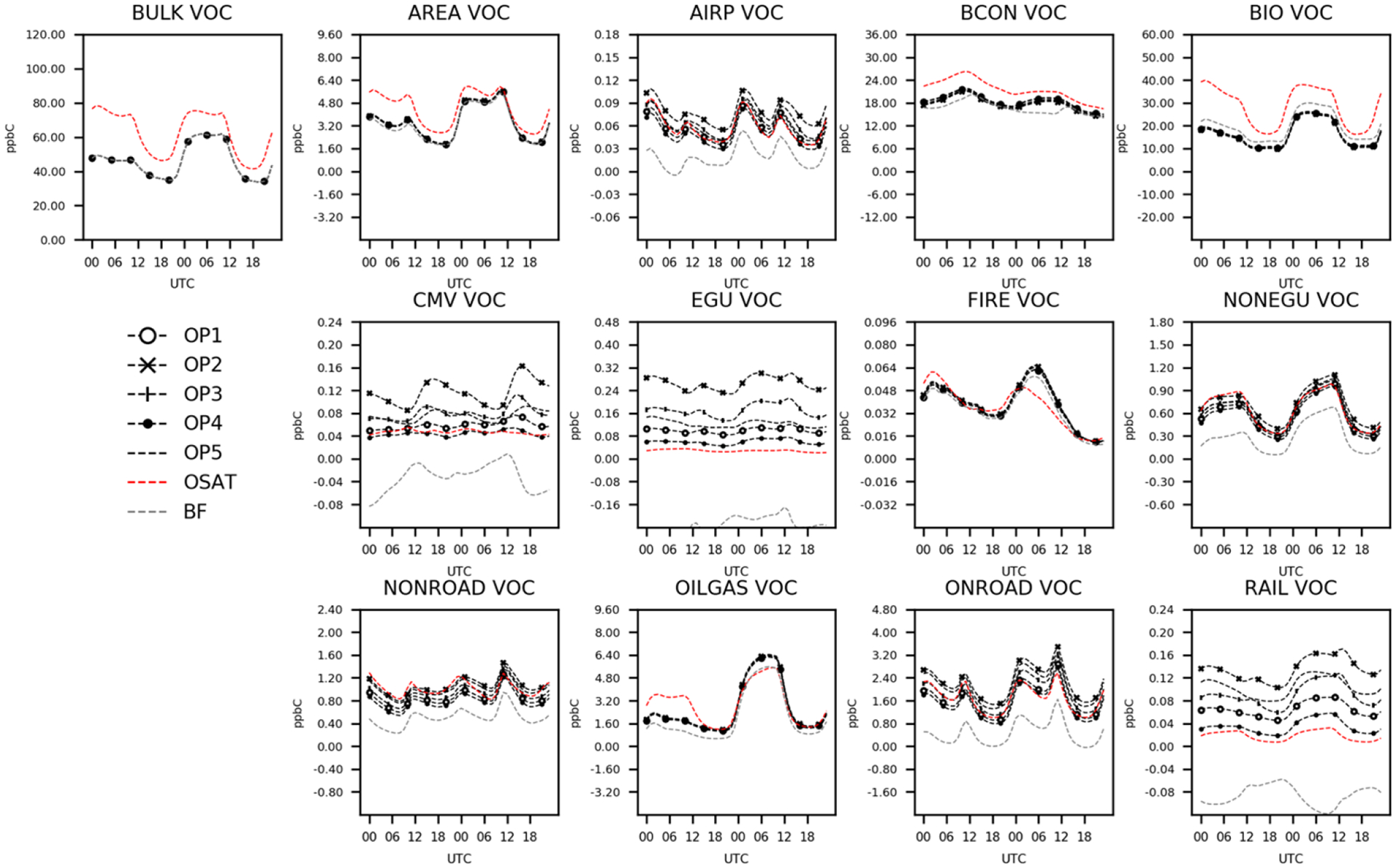
Total and attributed VOC concentrations to various sectors as a function of hour of day and apportionment technique.

Figure 5 illustrates the hourly variability of domain-averaged VOC concentrations and sector contributions. CAMx only gives pre-lumped VOC (Table 4) for OSAT outputs. For consistency, VOC for CMAQ ISAM and BF has also been carbon-weighted by summing all individual VOC species in CMAQ outputs using the same method as OSAT (Table 4). In Fig. 5, CAMx consistently simulates higher attribution to total VOC concentrations than CMAQ, with a maximum difference of 30 ppb. These larger CAMx VOC concentrations are also reflected in apportioned OSAT sectors, particularly those with substantial contributions, such as BCON and BIO. Given that the difference is present in the total concentration, this is unlikely caused by different source apportionment formulation between CMAQ and CAMx. As CAMx only gives pre-lumped VOC, it is challenging to compare individual VOC species between CMAQ and CAMx to explain this difference at current stage. Another possible reason for this could be that models have different internal treatments for advection and diffusion, which can impact surface-level concentrations and indirectly impact chemical reactions. The five ISAM options have comparable diurnal patterns for most sectors, with the exception of CMV, EGU, and RAIL; however, the magnitudes for these three sectors are relatively minor, which is consistent with earlier findings (Kwok et al., 2015). CMAQ-BF estimates notably lower sector contributions for VOCs, which is similar to O_3_ results (Fig. 4), with negative contributions for small sectors (e.g., CMV, EGU, and RAIL). Additional figures of other grouped nitrogen species tracked in Table 4 (e.g., RGN, HNO_3_, and NO_*y*_) can be found in the Supplement.

#### 4.2.2 Spatial distribution of source apportionment simulations

**Figure 6.**
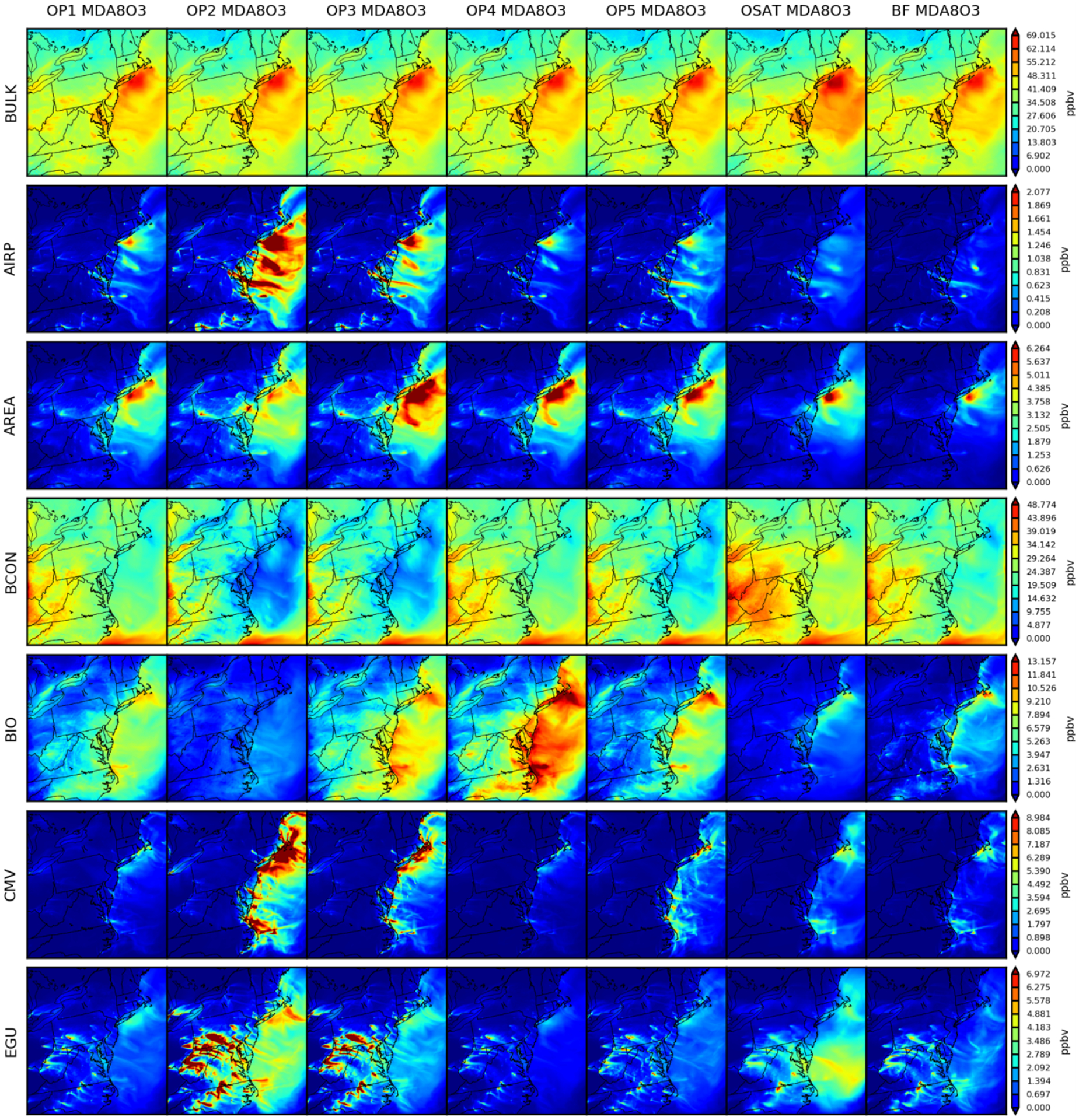

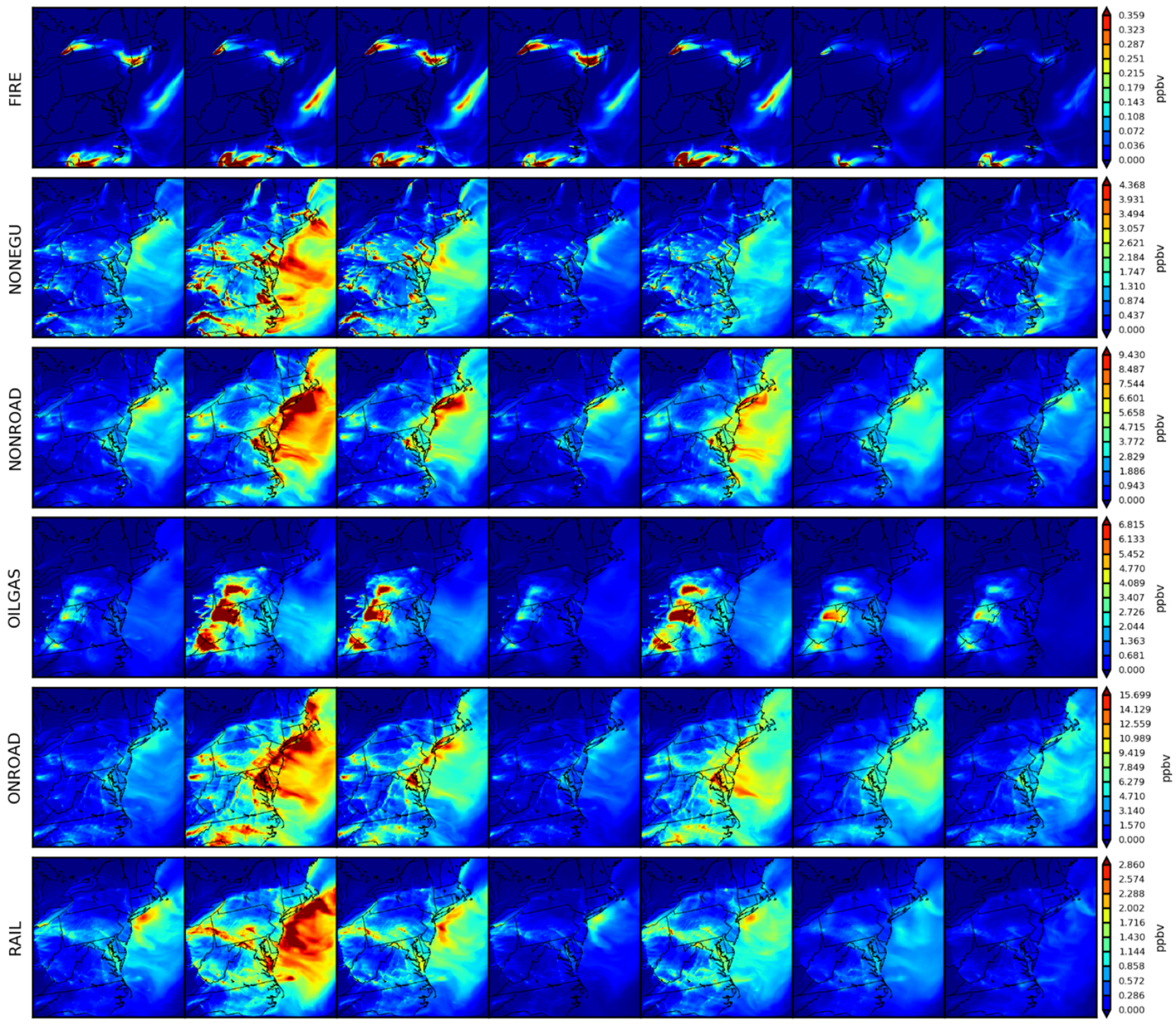
Spatial comparisons of seven simulations for 2 d averaged O_3_ (9 and 10 August).

**Figure 7.**
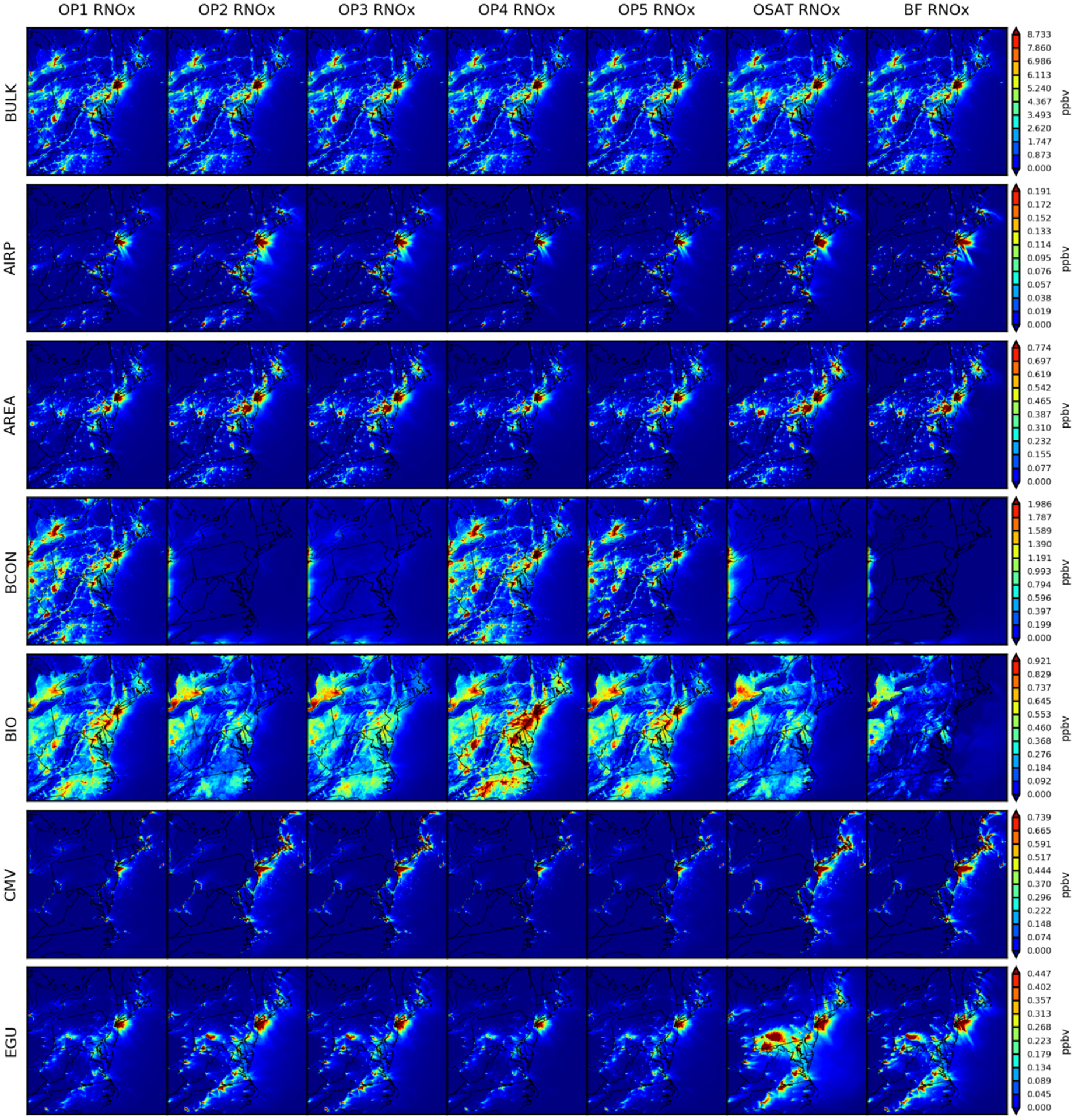

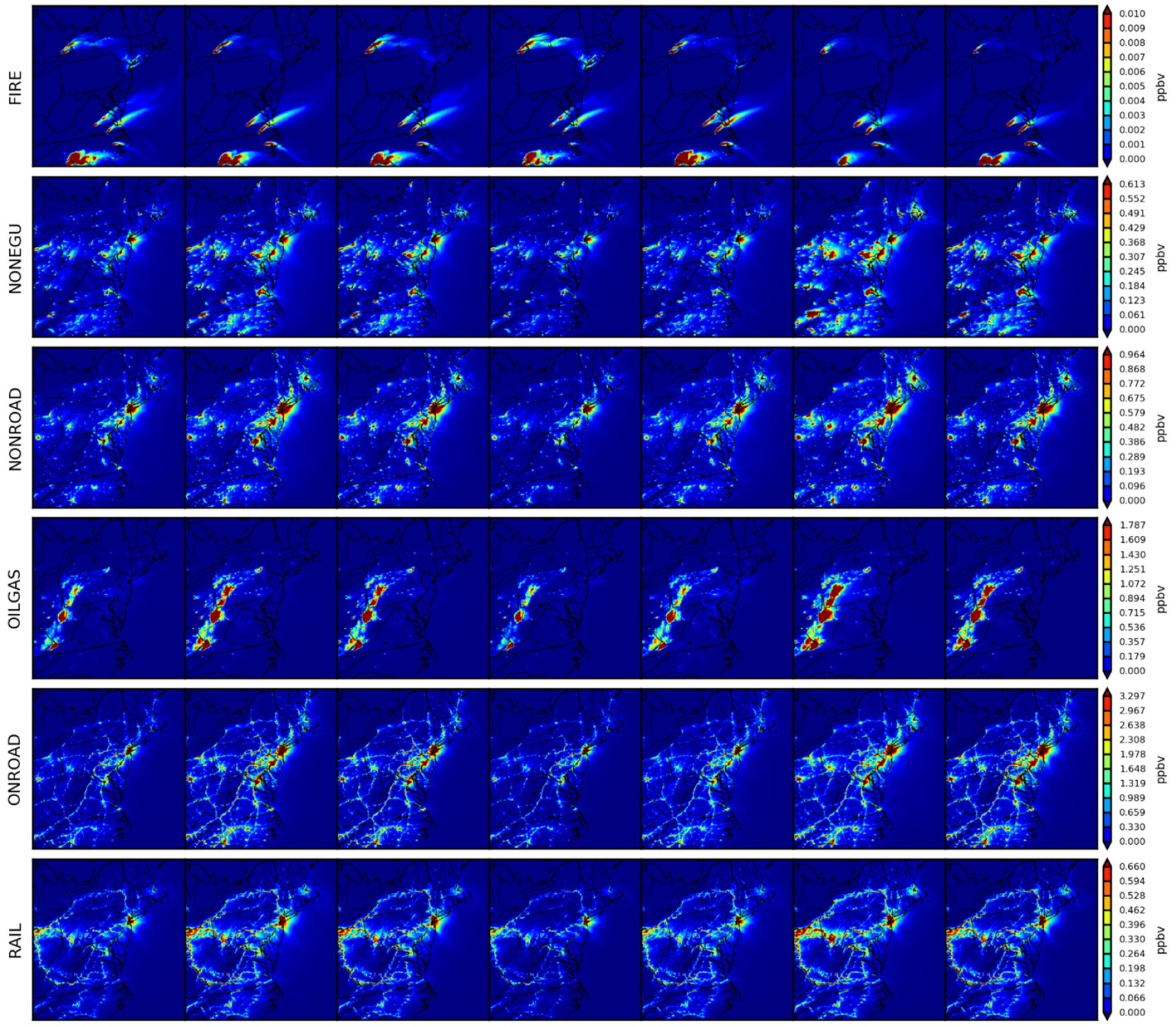
Spatial comparisons of seven simulations for 2 d averaged RNO_*x*_ (9 and 10 August).

**Figure 8.**
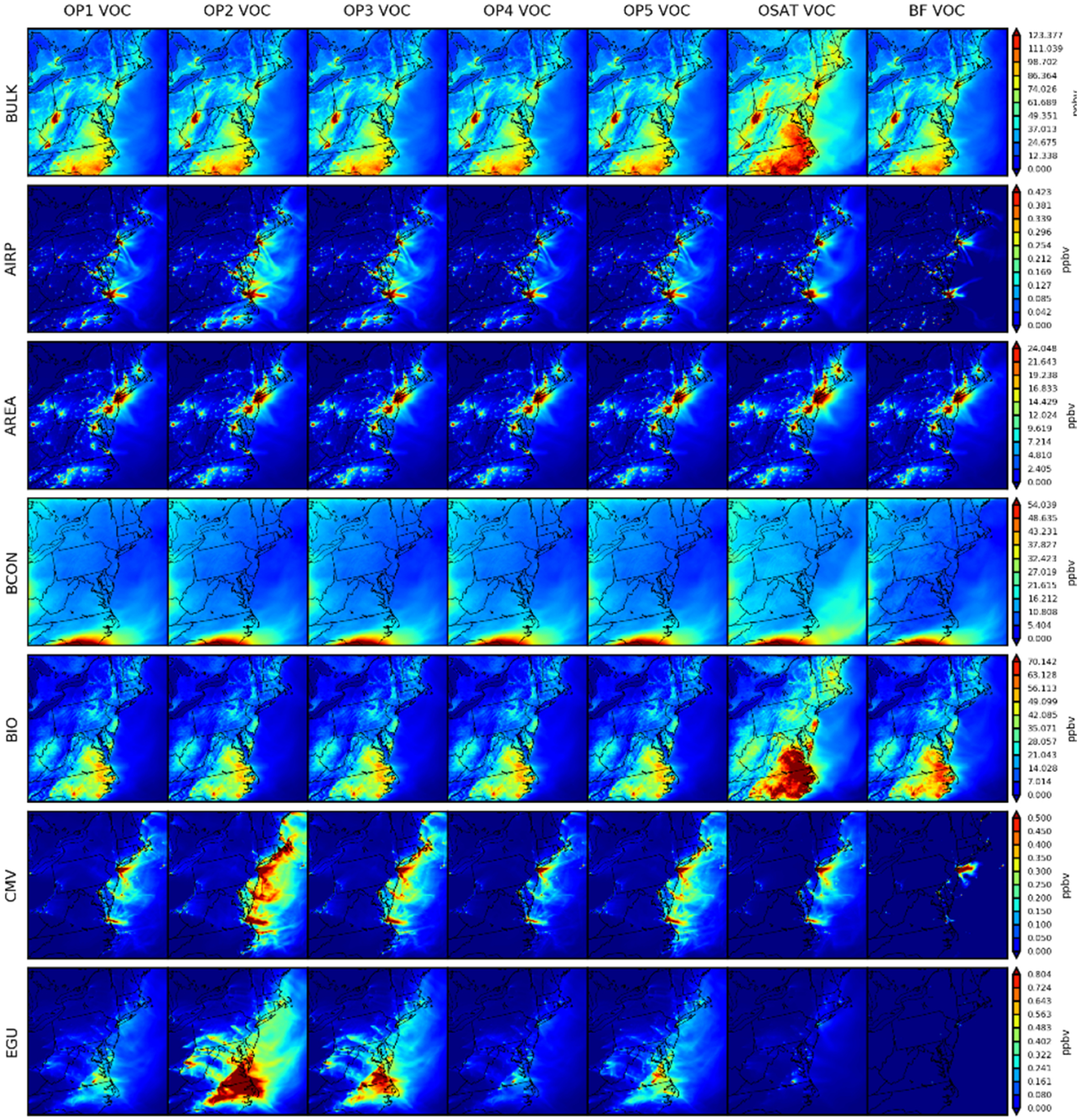

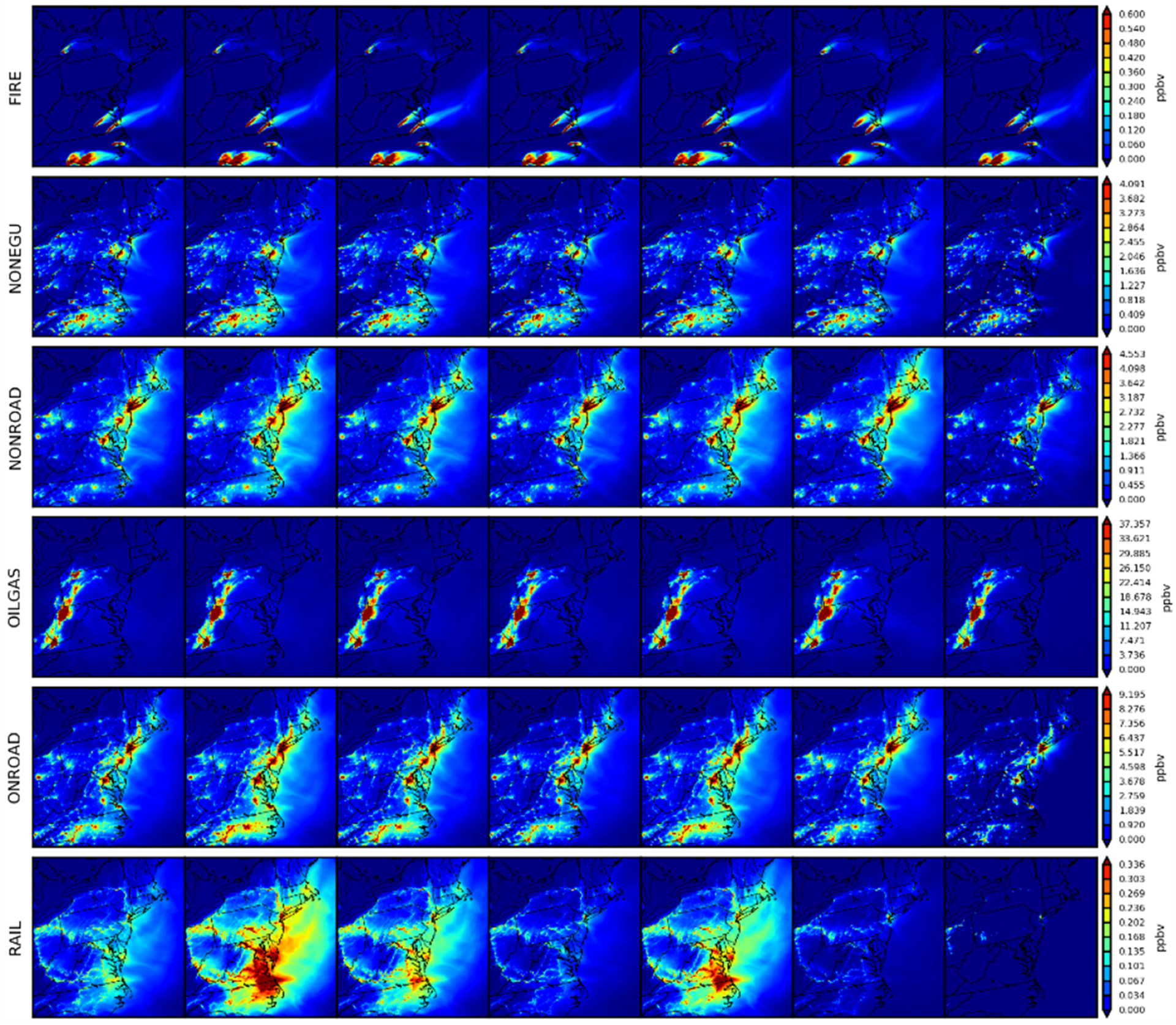
Spatial comparisons of seven simulations for 2 d averaged VOC (9 and 10 August).

Spatial patterns of total and sector contributions of MDA8 O_3_ (Fig. 6), RNO_*x*_ (Fig. 7) and VOC (Fig. 8) have been examined for the seven simulations. In Fig. 6, OSAT exhibits the same spatial distribution of MDA8 O_3_ total concentrations as other CMAQ-based simulations (OP1, OP2, OP3, OP4, OP5, and CMAQ-BF), with the exception of OSAT’s relatively high marine and offshore total concentrations (> 5 ppbv), which could be explained by the difference in planetary boundary layer dynamics or different marine chemistry configuration between the two parent models. CMAQ CB6R3 uses a rough parameterization for full marine halogen chemistry to destroy O_3_, depending only on land-use category and sunlight (Sarwar et al., 2015, 2019), whereas CAMx CB6R4 handles O_3_ depletion in the marine boundary more efficiently by including the 16 most important reactions of inorganic iodine (I-16b, Emery et al., 2016b). According to a sensitivity test conducted by Emery et al. (2016b), I-16b could reduce O_3_ depletions by 2–5 ppbv in comparison to full halogen chemistry. Regarding sector concentrations, the spatial distributions of seven simulations are comparable. They can all capture geographic contribution hot spots from each sector, although their magnitudes vary. OP2 stands out with fewer contributions from BIO than the other four ISAM options, and subsequently assigns larger concentrations to other sectors, particularly over east coastal regions, as shown in Figs. 3 and 6. Since OP2 assigns all products to sources with nitrogen reactants, the influence of reactants from biogenic sources is diminished, as intended.

Figure 7 depicts the associated outcomes of RNO_*x*_. Except for BCON, the seven simulations produce geographically and quantitatively consistent findings. From the spatial distributions, we can conclude that local sources govern RNO_*x*_ more than long-transported sources compared to O_3_. Anthropogenic RNO_*x*_ is either more concentrated in the urban areas (e.g., AREA, NONEGU, NONROAD), gasoline industry (OILGAS) and electric facilities (EGU) or along with transportation (e.g., AIRP, ONROAD, CMV and RAIL). Biogenic RNO_*x*_ is more prevalent in rural locations with vegetation. It should be noted that OP1, OP4, and OP5 show more BCON RNO_*x*_ across the entire domain because of the method used to assign products in nitrogen-related reactions (Sect. 2). OP1, OP4, and OP5 show local hotspots of RNO_*x*_ attributed to BCON. Since there is no physical reason to suspect hotspots over urban areas, we conclude that these contributions represent RNO_*x*_ attributed based on VOC or oxidants transported from the boundary. Figure 8 depicts the outcomes associated with VOC. Higher VOC concentrations from CAMx already shown in Fig. 5 are primarily from Virginia and North Carolina (OSAT bulk). As CMAQ and CAMx both use the same BEIS inventory data, the difference in total VOC concentrations may result from other differences between two models, like chemistry or deposition, accordingly leading to higher biogenic sources in CAMx (BIO). For the rest of the sectors, OSAT and ISAM options are fairly consistent except that the OP2 predicts more contributions from EGU, CMV, and RAIL. CMAQ-BF predicts consistently lower source contributions for MDA8 O_3_, RNO_*x*_, and VOC, as shown in Sect. 4.2.1. This yet again illustrates that brute force represents an integrated sensitivity while the OSAT and ISAM represent attribution at a point in the nonlinear chemical systems. Monthly averaged spatial maps for MDA8 O_3_, RNO_*x*_, and VOC are also included in Fig. S4a–c in the Supplement and show consistent results as 2 d averaged maps. This demonstrates that our case study is appropriate, efficiently selecting representative days as well as minimizing the uncertainties from parent models (CMAQ and CMAQ). Additional figures of other grouped nitrogen species tracked in Table 4 (e.g., RGN, HNO_3_ and NO_*y*_) can also be found in the Supplement.

## 5 Model simulation time

The CPU time required to complete a source apportionment simulation in a 3D AQM is an important consideration for usability. For a 4 km × 4 km simulation domain encompassing the northeastern US, the model run times for OSAT and ISAM are similar. Using 128 processors, base CMAQ (without ISAM) and CMAQ-ISAM simulations (11 source categories) are tested. Base CMAQ requires around 60 min per simulation day (24 h), whereas CMAQ-ISAM requires approximately 120 min. If the number of processors is increased to 256, the simulation time for CMAQ-ISAM can be reduced by 30 min, showing good scalability. It is worth noting that our CMAQ-ISAM simulations simultaneously track all additional species classes, such as sulfate, nitrate, ammonium, elemental carbon, organic carbon, and chloride. It would shorten simulation times if related species were only tracked for O_3_. Base CAMx (without OSAT) and CAMx OSAT are also tested with 128 processors, taking 37 and 67 min, respectively. CAMx also provides an optional tool for particles that can be simultaneously applied similarly to ISAM (PSAT, Yarwood et al., 2007). When additional pollutants are selected for tracking (e.g., sulfate, primary PM_2.5_ species) total simulation time will increase for both ISAM and OSAT and/or PSAT. CMAQ-BF speed is based on CMAQ base simulation (60 mind^−1^ × (1 base + 11 sources + 1 boundary condition + 1 initial condition 1 other) = 900 mind^−1^).

## 6 Discussions and conclusions

Source attribution approaches are generally intended to determine culpability of precursor emission sources to ambient pollutant concentrations. Source-based apportionment approaches such as ISAM and OSAT provide similar types of information, specifically an estimate of which sources or groups of sectors (e.g., a sector) contributed to the air quality measured or estimated at a particular location. The assumptions in each technique have implications for interpretations in the context of air quality management.

Source attribution of secondarily formed pollutants cannot be explicitly measured, which makes evaluation of source apportionment approaches challenging. Here, the ISAM approach was evaluated by (i) a comparison with a source apportionment approach implemented in a different photochemical modeling system and (ii) a comparison with a simple source sensitivity (brute-force difference) approach in the same modeling system that is most comparable to source apportionment in more linear systems and less useful when formation and transport are nonlinear. Further, this section notes qualitative consistency between the spatial nature of sector emission and the attribution of precursors and O_3_ as another method to generate confidence in these approaches.

In this study, multiple apportionment approach comparisons show common features but still reveal wide variations in predicted sector contribution and species dependency. The attribution to sources emitting NO_*x*_ and VOC is consistent with the spatial nature of these sources, which provides confidence in the approach. However, nitrogen species (e.g., NO_*x*_), for instance, are more sensitive to the choice of ISAM options than VOC. For example, although the attribution of NO_*x*_ to EGUs matches the location of these sources (e.g., New York urban area) for all ISAM options, OP1, OP4, and OP5 predict more BCON NO_*x*_. This is because the fast NO_*x*_ cycling process assigns anthropogenically emitted nitrogen species to other sources, as the original emitted source identity is not retained through these complex reactions. Further, sources entirely located offshore, such as commercial marine vessels, do not have culpability assigned to distant inland regions of the model domain. Most of the time, the amount of attribution to a certain sector depends on the number of emissions from that sector, how far away those emissions are, and whether the prevailing winds carried emissions from those places to the monitor or grid cell where air quality was predicted.

The five designed ISAM options maximize its flexibility, particularly for modeling source apportionment of O_3_ and its precursors, but the choice of option depends on target species. Among all ISAM options, the OP5 option, after making the assignment decision based on the ratio of PH_2_O_2_ to PHNO_3_, is expected to predict generally similar spatial and temporal patterns for O_3_ to the OSAT source apportionment approach implemented in CAMx. However, it still shows disparity for some sectors (e.g., biogenic sectors for O_3_). This result may be because of the OSAT formulation, which differs from the ISAM options presented here. The OP5 option was also similar to brute-force sensitivity estimates predicted in CMAQ with the exception of source groups that dominate regional emissions or O_3_, such as biogenic VOC and O_3_ introduced into the model through boundary inflow. In those situations, it is not reasonable to expect a source sensitivity approach to provide a useful comparison for source attribution given the highly nonlinear change in atmospheric chemistry. After assigning products to sources emitting nitrogen reactants, the OP2 option can predict results of RNO_*x*_ attributions that are more comparable to OSAT and BF. It demonstrated that the OP2 works better for RNO_*x*_ because it makes it easier to find the original source and lessens the effect of other sources when these species are cycling quickly through an integrated chemical reaction system. Unlike O_3_ and RNO_*x*_, the VOC contribution for the majority of source categories depends very little on the ISAM option. We expect that the user will use OP5 for O_3_ and OP2 for RNO_*x*_, but this is not a firm suggestion. In turn, we give the user this flexibility so that ISAM can be used for a wide range of purposes.

By comparing the multiple approaches in the northeastern US, we found that both OSAT and ISAM attribute the majority of O_3_ and NO_*x*_ contributions to boundary, mobile, and biogenic sources, whereas the top three VOC contributions are attributed to biogenic, boundary, and area sources. However, comparisons of OSAT and ISAM have some limits, especially when they are under the two different parent models, CAMx and CMAQ. Although we have put efforts into diminishing the differences between the two models by making most configuration options as similar as possible, some inevitable uncertainties cannot be eliminated at the current stage of this study (e.g., an imperfect match of chemical mechanisms or different internal treatments for advection, diffusion, and deposition processes). Further, it is also worthwhile to note that our results in this study are based on limited duration and specific regions, and they may not comprehensively reflect all situations. Given that the source attribution of secondary pollutants cannot be explicitly measured, these inter-comparisons between ISAM and OSAT are still useful for reference. We continue to need further efforts that combine field experiment studies and model evaluations for longer terms and multiple regions to better understand source attribution given the highly nonlinear change in nature of O_3_-NO_*x*_ chemistry.

## Supplementary Material

Supplement1

## Figures and Tables

**Table 1.** Expanded CMAQ-ISAM options.

CMAQ ISAM option	Reaction product source identity assignment	Representative CB6R3* species
ISAM-OP1	Proportional to stoichiometry of all reactants.	All tracked model species
ISAM-OP2	Proportional to stoichiometry of nitrogen containing reactants, otherwise same as ISAM-OP1.	NO, NO_2_, NO_3_, HONO, HNO_3_, N_2_O_5_, ANO_3_
ISAM-OP3	Proportional to stoichiometry of key O_3_ chemistry reactants (reactive VOCs, radicals, nitrogen species), otherwise same as ISAM-OP1.	NO, NO_2_, NO_3_, HONO, HNO_3_, N_2_O_5_, ANO_3_, ALD_2_, ALDX, FORM, ACET, KET, XO_2_, XO_2_H, ISO_2_, C_2_O_3_, CXO_3_
ISAM-OP4	Proportional to stoichiometry of VOC and radical containing reactants, otherwise same as ISAM-OP1.	ALD_2_, ALDX, FORM, ACET, KET, XO_2_, XO_2_H, ISO_2_, C_2_O_3_, CXO_3_
ISAM-OP5	According to the ratio of PH_2_O_2_ to PHNO_3_ if O_3_ chemistry reactants present, otherwise same as ISAM-OP1.	NO_*x*_-limited: NO, NO_2_, NO_3_, HONO, HNO_3_, N_2_O_5_, ANO_3_ VOC-limited: ALD_2_, ALDX, FORM, ACET, KET, XO_2_, XO_2_H, ISO_2_, C_2_O_3_, CXO_3_

* Species are based on CB6R3 and may vary based on different chemical mechanisms implemented in CMAQ. Details can be found in SA_DEFN.F in the CMAQ source code.

**Table 2.** CMAQ and CAMx model configurations

Model option	CMAQ	CAMx
Model version	Version 5.3.2	Version 7.10
Horizontal resolution	4 km	4 km
Vertical layers	35	35
Meteorology	WRF3.8	WRF3.8
Anthropogenic emissions	2016 NEI version 1^a^	2016 NEI version 1^b^
Biogenic emissions	BEIS^c^	BEIS^c^
BC/IC	12 km US CONUS	12 km US CONUS
Gas phase chemistry	CB6R3	CB6R4
Chemistry solver	EBI	EBI
Aerosol dynamics and chemistry	AERO7/ISORROPIA	SOAP/ISORROPIA
Horizontal advection	PPM	PPM
Vertical advection	PPM	Emery et al. (2011) ^d^
Horizontal diffusion	Implicit^e^	Explicit simultaneous 2-D solver
Vertical diffusion	ACM2^f^	Based on ACM2^g^
Gas deposition	Pleim and Ran (2011)	Zhang et al. (2003)
Particle deposition	Shu et al. (2022)	Zhang et al. (2001)
Source apportionment	ISAM	OSAT3

a EGUs were based on continuous emissions monitoring data from 2018 where available. On-road emissions were projected to 2018.

b CAMx EGU and on road were identically processed as CMAQ.

c BELD v4.1 vegetation data for biogenic emissions, and the BEIS version is 3.61.

d Backward Euler (time) hybrid centered and upstream (space) solver.

e Horizontal diffusion fluxes for transported pollutants were parameterized using eddy diffusion theory. The horizontal diffusivity coefficients were formulated using the approach of Smagorinsky (1963).

f KZMIN was turned on in CMAQ as default.

g Vertical diffusivity coefficients were calculated with Yonsei University (YSU) bulk boundary layer scheme (Hong et al., 2006) and were adjusted with the KVPATCH, which is comparable to the KZMIN approach in CMAQ.

**Table 3.** Total emissions from each sector for the 4 km northeastern US domain (month of August 2018).

Sector	Tons per month		Percent of total (%)	
	NO_*x*_	VOC	NO_*x*_	VOC
AIRP	2536	1198	1.6	0.1
AREA	10 617	95 434	6.8	8.7
BIO	8721	895 829	5.5	81.6
CMV	6262	684	4.0	0.1
EGU	22 458	791	14.3	0.1
FIRE	400	5007	0.3	0.5
NONEGU	15 020	11 323	9.6	1.0
NONROAD	23 958	33 561	15.2	3.1
OILGAS	11 053	22 526	7.0	2.1
ONROAD	49 361	30 578	31.4	2.8
RAIL	6847	318	4.4	0.0
Total	157 233	1 097 247	100	100

**Table 4.** Tracked species classes between ISAM and OSAT.

OSAT	ISAM
O_3_	O_3_
RGN = NO_2_ + NO_3_ + 2 × N_2_O_5_ + INO_3_	^1^ RGN = NO_2_ + NO_3_ + 2 × N_2_O_5_
NIT = NO + HONO	NIT = NO + HONO
TPN = PAN + PNA + PANX + OPAN + INTR	^2^ TPN = PAN + PNA + PANX + INTR
NTR = NTR_1_ + NTR_2_ + CRON	^3^ NTR = NTR_1_ + NTR_2_
HNO_3_	HNO_3_
RNO_*x*_ = RGN + NIT	RNO_*x*_ = RGN + NIT
NO_*y*_ = RGN + NIT + TPN + NTR + HNO_3_	NO_*y*_ = RGN + NIT + TPN + NTR + HNO_3_
^4^ VOC = 1.0 × PAR + 1.0 × MEOH + 1.0 × FORM + 1.0 × KET + 2.0 × ETHA + 2.0 × ETOH + 2.0 × ETH + 2.0 × OLE + 2.0 × ALD_2_ + 2.0 × ALDX + 2.0 × ETHY + 3.0 × PRPA + 3.0 × ACET + 4.0 × IOLE + 5.0 × ISOP + 6.0 × BENZ + 7.0 × TOL + 8.0 × XYL + 10.0 × TERP	VOC = 1.0 × PAR + 1.0 × MEOH + 1.0 × FORM + 1.0 × KET + 2.0 × ETHA + 2.0 × ETOH + 2.0 × ETH + 2.0 × OLE + 2.0 × ALD_2_ + 2.0 × ALDX + 2.0 × ETHY + 3.0 × PRPA + 3.0 × ACET + 4.0 × IOLE + 5.0 × ISOP + 6.0 × BENZ + 7.0 × TOL + 8.0 × XYL + 10.0 × TERP

1 ISAM does not track INO_3_.

2 ISAM does not track OPAN.

3 ISAM does not track CRON.

4 OSAT VOC has been pre-calculated as an equation in Table 4.

**Table 5.** Model performance summary at paired AQS surface monitoring sites. (monthly episode).

Species	Model	Number of observations	MB^a^	NMB^b^	RMSE^c^	*R* ^2 d^
Hourly NO	CMAQ	72 987	−1.05	−44.50	6.24	0.07
	CAMx	72 987	−1.23	−52.25	6.39	0.05
Hourly NO_2_	CMAQ	61 987	0.64	10.21	6.39	0.32
	CAMx	61 987	1.86	29.78	7.57	0.28
Hourly O_3_	CMAQ	232 768	6.49	23.11	11.73	0.59
	CAMx	232 768	7.99	28.47	14.46	0.42
MDA8 O_3_	CMAQ	9409	5.30	12.80	8.23	0.64
	CAMx	9409	4.18	10.09	9.26	0.58

a Mean bias is $\text{MB}=\frac{1}{N}\sum M_{i}-O_{i}$. MB ranges from negative infinity to positive infinity with 0 indicating unbiased data, and the unit here is ppbv.

b Normalized mean bias is $\text{NMB}=\frac{1}{N}\sum \frac{M_{i}-O_{i}}{O_{i}}$, and this ranges from negative 1 to positive infinity with 0 indicating unbiased data. The values shown in the table were multiplied by 100.

c Root-mean-square error is $\text{RMSE}=\sqrt{\frac{1}{n}\Sigma_{i=1}^{n}{\left(M_{i}-O_{i}\right)}^{2}}$, and this is the standard deviation of the prediction errors.

d $R^{2}={\left\{\frac{\sum \left(O_{i}-\bar{O}\right)\left(M_{i}-\bar{M}\right)}{\sqrt{\sum {\left(O_{i}-\bar{O}\right)}^{2}\sum {\left(M_{i}-\bar{M}\right)}^{2}}}\right\}}^{2}$. *R*^2^ ranges from 0 to 1, with 1 indicating perfect correlation and 0 indicating an uncorrelated relationship.

**Table 6.** Model performance summary at paired AQS surface monitoring sites. (2 d case study episode)

Species	Model	Number of observations	MB^a^	NMB^b^	RMSE^c^	*R* ^2 d^
Hourly NO	CMAQ	4264	−1.15	−48.30	6.44	0.05
	CAMx	4264	−1.38	−58.14	6.57	0.04
Hourly NO_2_	CMAQ	3612	0.15	2.20	6.83	0.28
	CAMx	3612	0.83	11.88	7.51	0.25
Hourly O_3_	CMAQ	13 486	4.67	15.06	10.88	0.61
	CAMx	13 486	7.02	22.65	13.26	0.49
MDA8 O_3_	CMAQ	567	2.75	6.00	6.28	0.62
	CAMx	567	2.80	6.10	6.95	0.63

a Mean bias is $\text{MB}=\frac{1}{N}\sum M_{i}-O_{i}$. MB ranges from negative infinity to positive infinity with 0 indicating unbiased data, and the unit here is ppbv.

b Normalized mean bias is $\text{NMB}=\frac{1}{N}\sum \frac{M_{i}-O_{i}}{O_{i}}$, and this ranges from negative 1 to positive infinity with 0 indicating unbiased data. The values shown in the table were multiplied by 100.

c Root-mean-square error is $\text{RMSE}=\sqrt{\frac{1}{n}\Sigma_{i=1}^{n}{\left(M_{i}-O_{i}\right)}^{2}}$, and this is the standard deviation of the prediction errors.

d $R^{2}={\left\{\frac{\sum \left(O_{i}-\bar{O}\right)\left(M_{i}-\bar{M}\right)}{\sqrt{\sum {\left(O_{i}-\bar{O}\right)}^{2}\sum {\left(M_{i}-\bar{M}\right)}^{2}}}\right\}}^{2}$. *R*^2^ ranges from 0 to 1, with 1 indicating perfect correlation and 0 indicating an uncorrelated relationship.

## Data Availability

The raw observation data used are available from the sources identified in Sect. 3 (https://www.epa.gov/aqs, U.S. EPA, 2022c), while the post-processed observation data are available upon request. The CMAQ model data utilized are available upon request as well. Please contact the corresponding author to request any data related to this work.
