# Supplementary material for: Comparison of ozone formation attribution techniques in the northeastern United States
Source: Geosci Model Dev. Author manuscript; Available in PMC 2025 Jan 2. (PMC11694848; doi:10.5194/gmd-16-2303-2023)
Supplement: Supplement1 [file NIHMS2032979-supplement-Supplement1.pdf]

Supplement of Geosci. Model Dev., 16, 2303–2322, 2023
https://doi.org/10.5194/gmd-16-2303-2023-supplement
© Author(s) 2023. CC BY 4.0 License.

Geoscientific Model Development
Open Access

Supplement of

Comparison of ozone formation attribution techniques in the northeastern United States

Qian Shu et al.

Correspondence to: Sergey L. Napelenok (sergey.napelenok@epa.gov)

The copyright of individual parts of the supplement might differ from the article licence.

Contents:

Section S1: Criteria to select representative days for ISAM comparison

Section S2: Additional evaluations of O_3, NO and NO_2

Section S3: Temporal variations of sector contributions for additional tracked species in Table 4

Section S4: Spatial distribution of source apportionment simulations for monthly averaged MDA8 O_3, RNO_x and VOC

Section S5: Spatial distribution of source apportionment simulations for additional two-day averaged tracked species in Table 4

Section S6: Temporal and spatial averaged source contributions for MDA8 O_3, RNO_x and VOC

Table S1: Criteria to select representative days for ISAM comparison

Table S2(a): Domain-wide two-day mean percentage contributions (%) for MDA8 O_3, RNO_x, and VOC

Table S2(b): Domain-wide two-day mean absolute concentration contributions (ppb) for MDA8 O_3, RNO_x, and VOC

Fig. S1: observed site-averaged daily mean (a) O_3, (b) NO and (c) NO_2 and corresponding mean biases predicted by CMAQ and CAMx over paired AQS sites for the entire episode

Fig. S2: Two-day averaged observed (a) O_3, (b) NO and (c) NO_2 over paired sites for northeast US domain and its corresponding mean biases predicted by CMAQ and CAMx for selected case study

Fig. S3: Comparisons of hourly variations of (a) RGN, (b) NIT, (c) TPN, (d) NTR, (e) HNO_3 and (f) NO_y concentrations among seven source apportionment simulations (OP1 to OP5, OSAT, CMAQ-BF) for bulk mixing ratios and selected sector contributions

Fig. S4: Spatial comparisons of seven simulations for monthly averaged (a) MDA8 O_3, (b) RNO_x and (c) VOC (07/29-08/30)

Fig. S5: Spatial comparisons of seven simulations for two-day averaged (a) RGN, (b) NIT, (c) TPN, (d) NTR, (e) HNO_3, (f) NO_y and (g) O_3 (08/09 and 08/10)

Fig. S6: Two-day averaged domain-wide contributions of (a) MDA8 O_3, (b) RNO_x, and (c) VOC from each sector for seven source apportionment simulations (OP1 to OP5, OSAT, CMAQ-BF)

S1. Criteria to select representative days for ISAM comparison

Because there are still underlying process uncertainties that cannot be constrained, even when identical model inputs are used, the outputs of ISAM and OSAT might be impacted by their parent models (CMAQ and CAMx). We established criteria to choose representative days for ISAM and OSAT comparison based on the performance of their parent models rather than comparing them throughout the entire simulation period to reduce the difference that may be brought on by their parent models. We initially set the correlation relationship (R^2) criteria to be above 0.7 to ensure that the performance of the CMAQ and the CAMx is comparable. Next, we

assess the mean bias (MB) of MDA8 O_3 for every day to choose the days on which both models have the lowest MB for predicted MDA8 O_3. Table S1 contains a summary of the metrics.

Table S1. Criteria to select representative days for ISAM comparison.

Day	R^2	CMAQ MB (ppbv)	CAMx MB (ppbv)
7/29/18	0.54	1.94	2.13
7/30/18	0.5	3.97	1.08
7/31/18	0.6	4.75	2.88
8/1/18	0.4	5.02	-1.64
8/2/18	0.67	5.86	-1.13
8/3/18	0.73	8.34	0.8
8/4/18	0.79	7.84	7.19
8/5/18	0.7	9.68	13
8/6/18	0.74	9.25	13.32
8/7/18	0.67	8.15	7.27
8/8/18	0.57	6.06	2.52
8/9/18	0.7	3.09	2.99
8/10/18	0.78	2.42	2.61
8/11/18	0.5	6.45	4.22
8/12/18	0.62	6.53	5.87
8/13/18	0.22	6.91	1.99
8/14/18	0.07	4.99	1.77
8/15/18	0.69	2.95	5.09
8/16/18	0.78	4.29	5.89
8/17/18	0.75	6.56	5.07
8/18/18	0.48	7.19	0.73
8/19/18	0.73	7.3	5.64
8/20/18	0.48	9.45	7.42
8/21/18	0.3	6.79	2.21
8/22/18	0.48	6.56	3.56
8/23/18	0.62	4.18	5.14
8/24/18	0.4	0.79	3.98
8/25/18	0.33	0.75	3.71
8/26/18	0.34	3.8	4.05
8/27/18	0.7	3.27	4.53
8/28/18	0.82	1.32	5.72
8/29/18	0.79	2.69	4.34
8/30/18	0.68	6.26	4.89

S2. Additional evaluations of O_3, NO and NO_2

Figure S1(a) shows that the overestimation of daily mean O_3 by CMAQ and CAMx is more than that of MDA8 O_3, and the discrepancy between the two models continues to grow. It reveals that both models overestimate nighttime O_3, with CAMx predicting more than CMAQ, which could be attributed to the underestimation of O_3 titration by NO (Bessagnet et al., 2016; Sharma et al., 2017; Pay et al., 2019). Figure S1(b) shows that both models underestimate daily mean NO, with CAMx predicting less than CMAQ. In contrast, Figure S1(c) exhibits two models' overestimations of daily mean NO_2 but CAMx predicts higher NO_2 than CMAQ.

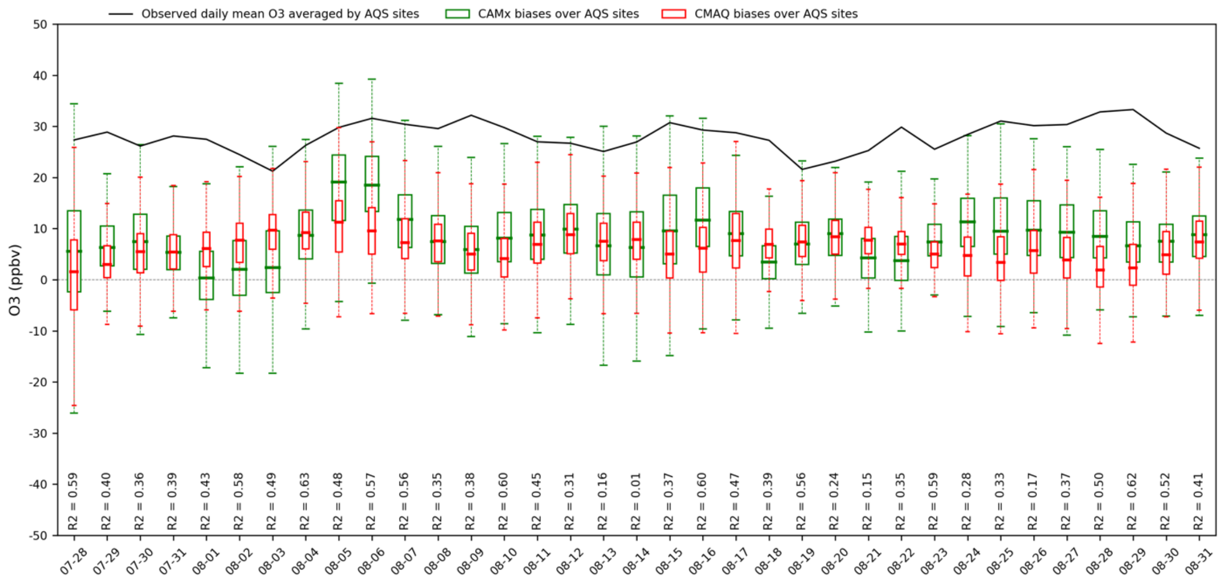

Fig. S1(a) observed site-averaged daily mean O_3 and its corresponding biases predicted by CMAQ and CAMx over paired AQS sites for the entire episode. R^2 shows correlation relationship between CMAQ and CAMx.

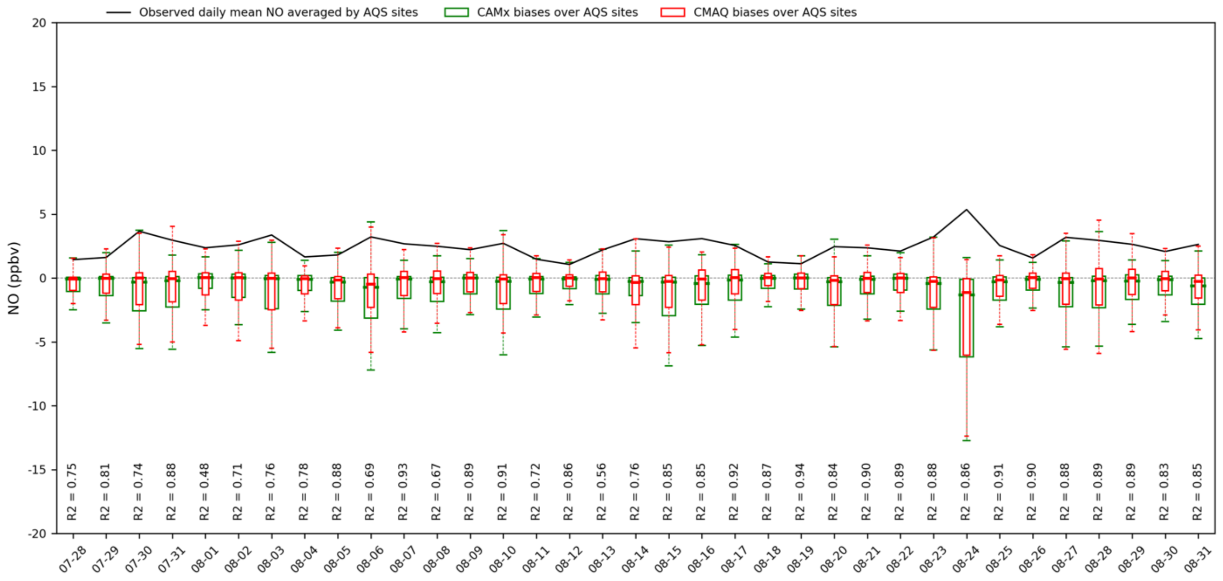

Fig. S1(b) observed site-averaged daily mean NO and its corresponding biases predicted by CMAQ and CAMx over paired AQS sites for the entire episode. R^2 shows correlation relationship between CMAQ and CAMx.

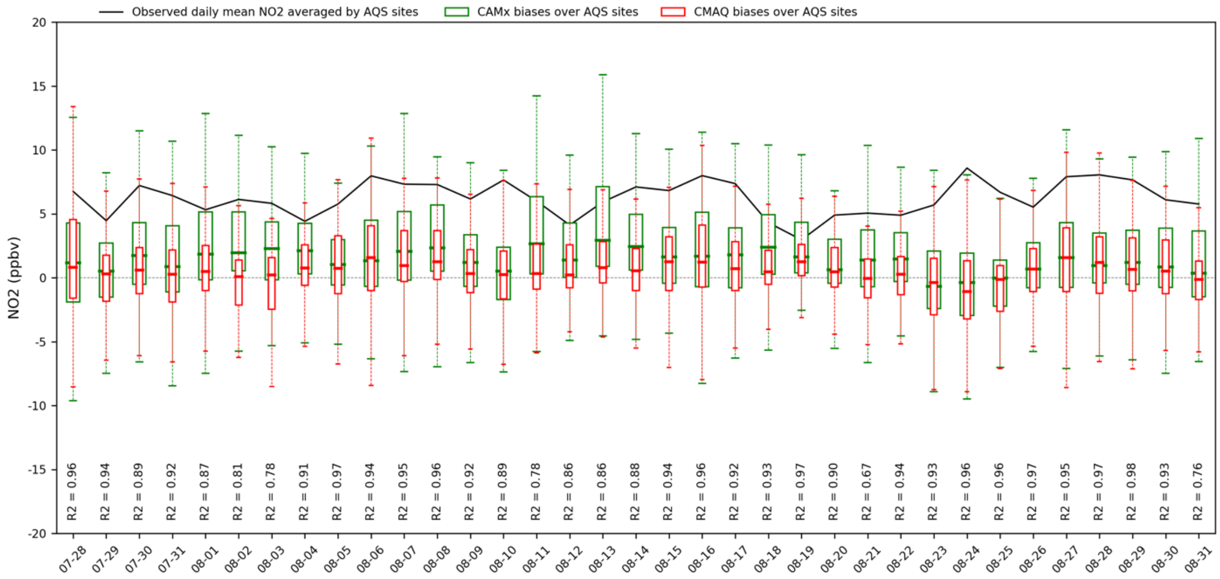

Fig. S1(c) observed site-averaged daily mean NO_2 and its corresponding biases predicted by CMAQ and CAMx over paired AQS sites for the entire episode. R^2 shows correlation relationship between CMAQ and CAMx.

Figure S2 spatially plots two-day averaged observed (a) MDA8 O_3, (b) NO and (c) NO_2 over paired sites for the northeast US domain and the corresponding mean biases predicted by CMAQ and CAMx for selected case study.

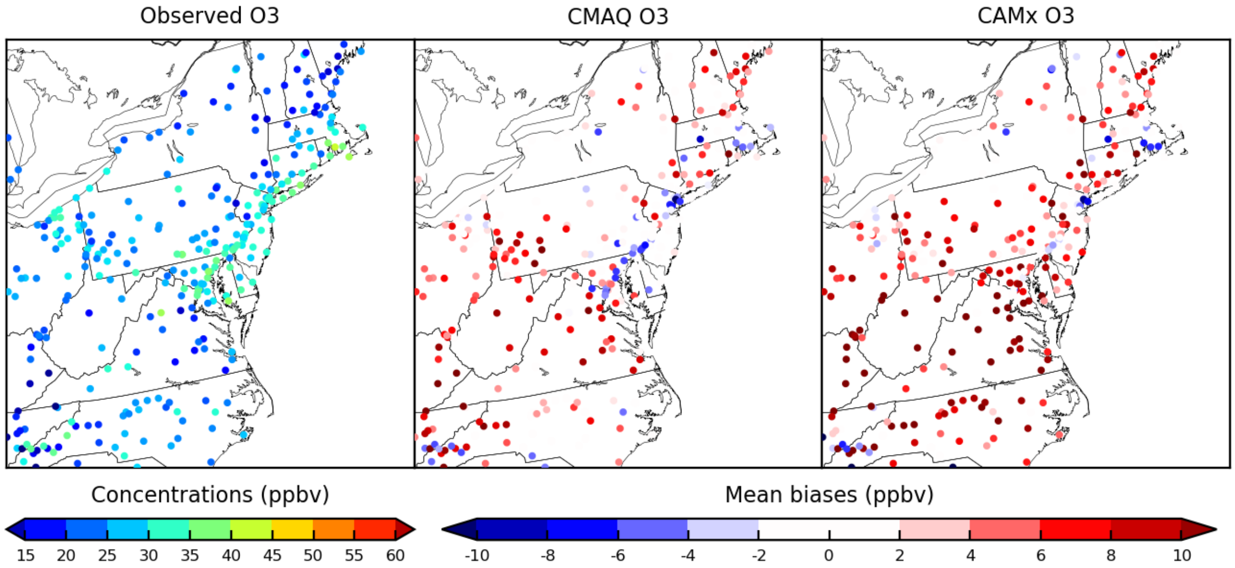

Fig. S2(a) Two-day averaged observed O_3 over paired sites for northeast US domain and its corresponding mean biases predicted by CMAQ and CAMx for selected case study.

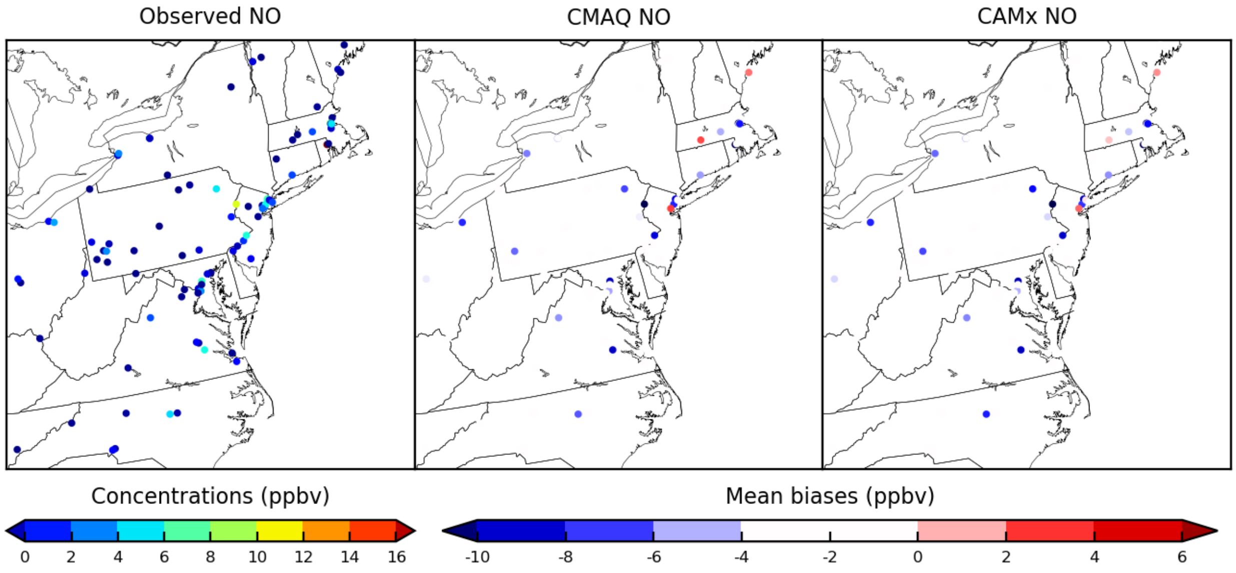

Fig. S2(b) Two-day averaged observed NO over paired sites for northeast US domain and its corresponding mean biases predicted by CMAQ and CAMx for selected case study.

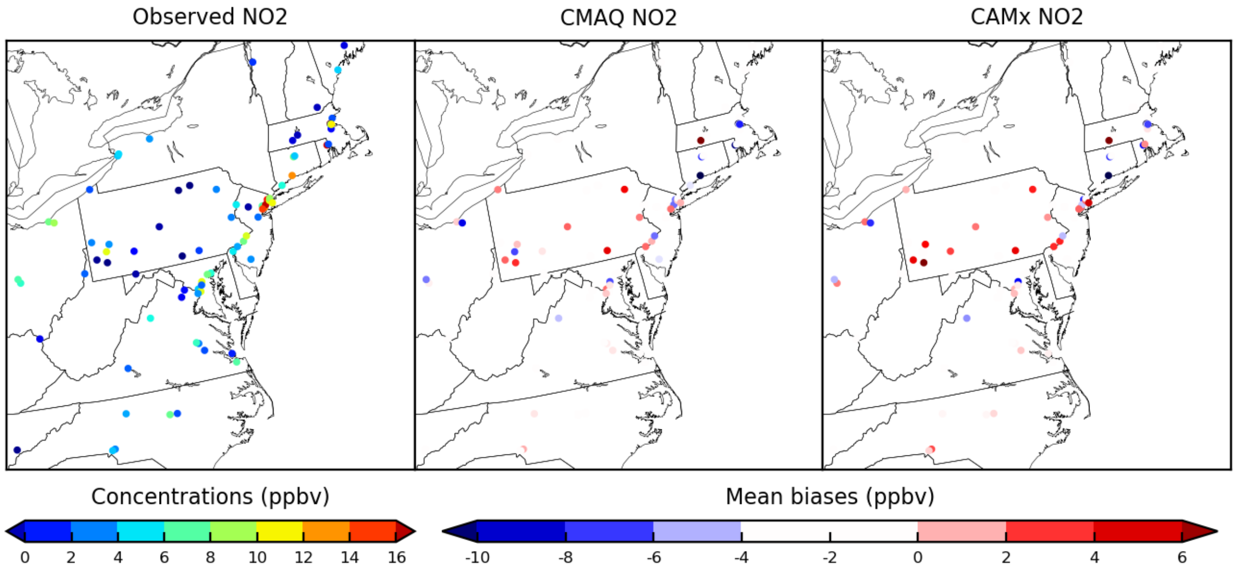

Fig. S2(c) Two-day averaged observed NO2 over paired sites for northeast US domain and its corresponding mean biases predicted by CMAQ and CAMx for selected case study.

S3. Temporal variations of sector contributions for additional tracked species in Table 4

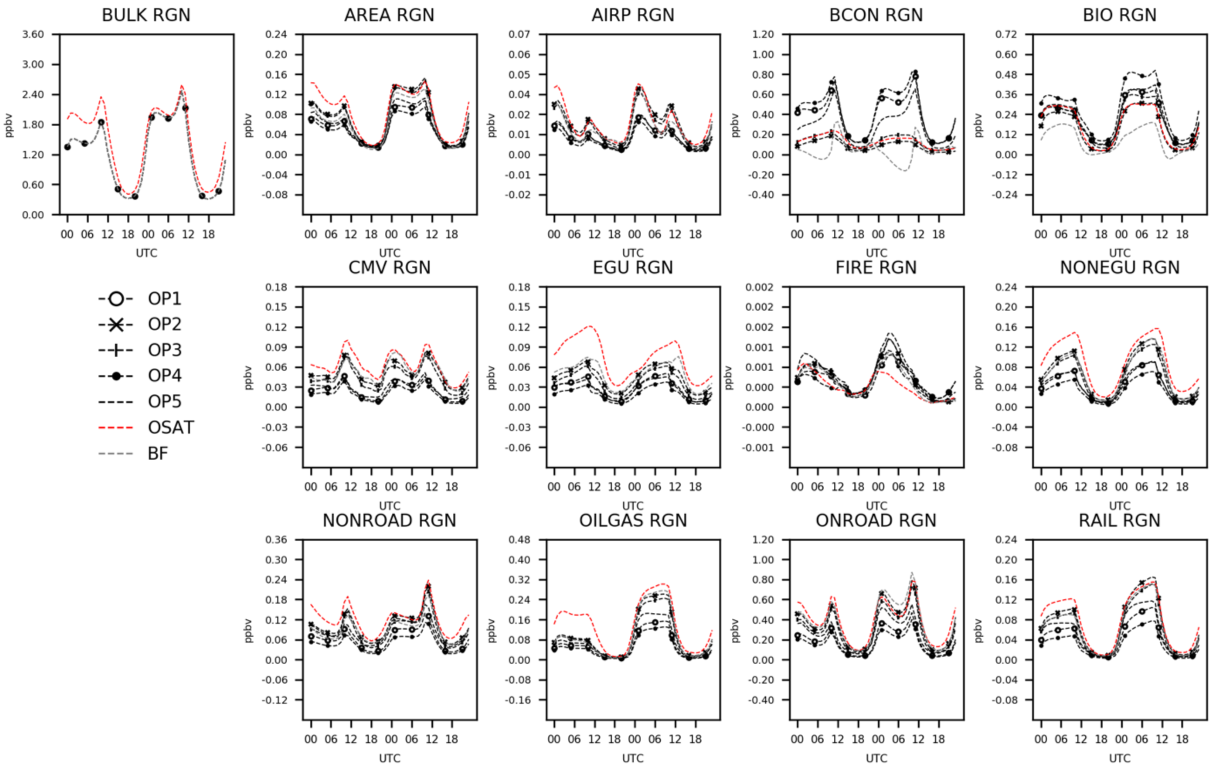

Fig. S3(a) Comparisons of hourly variations of RGN concentrations among seven source apportionment simulations (OP1 to OP5, OSAT, CMAQ-BF) for bulk mixing ratios and selected sector contributions.

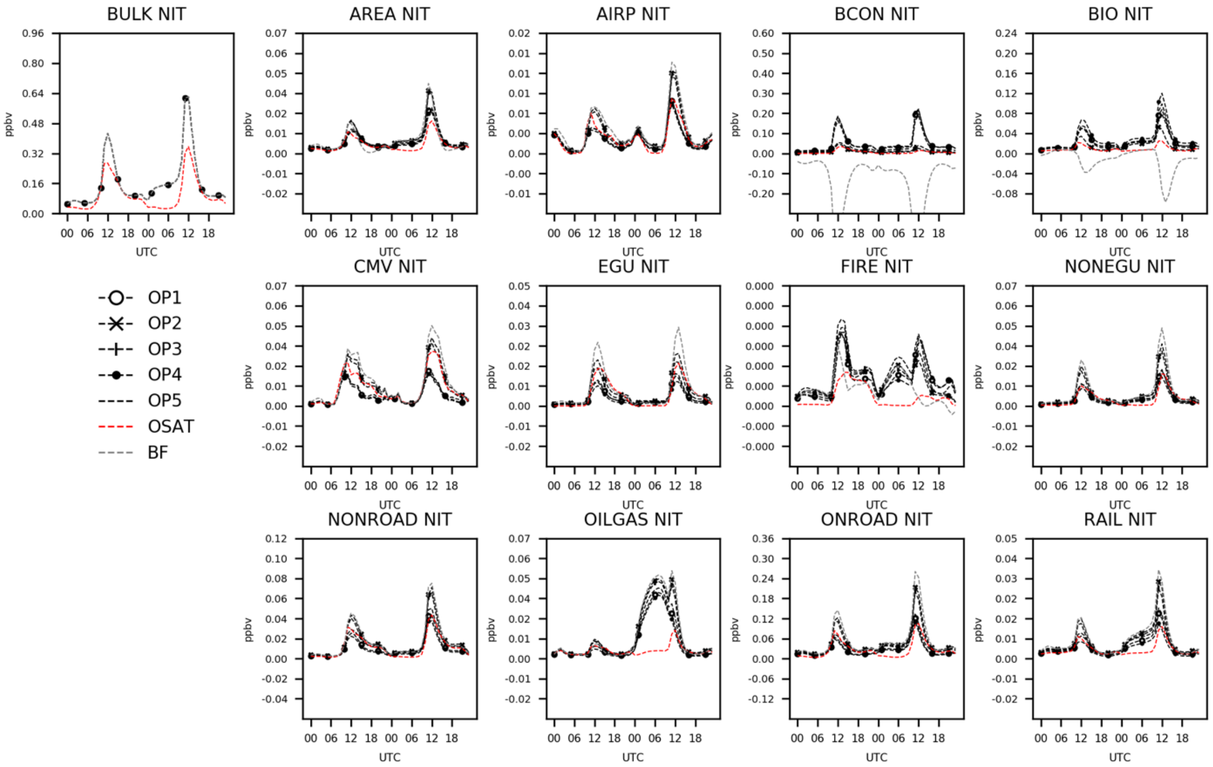

Fig. S3(b) Comparisons of hourly variations of NIT concentrations among seven source apportionment simulations (OP1 to OP5, OSAT, CMAQ-BF) for bulk mixing ratios and selected sector contributions.

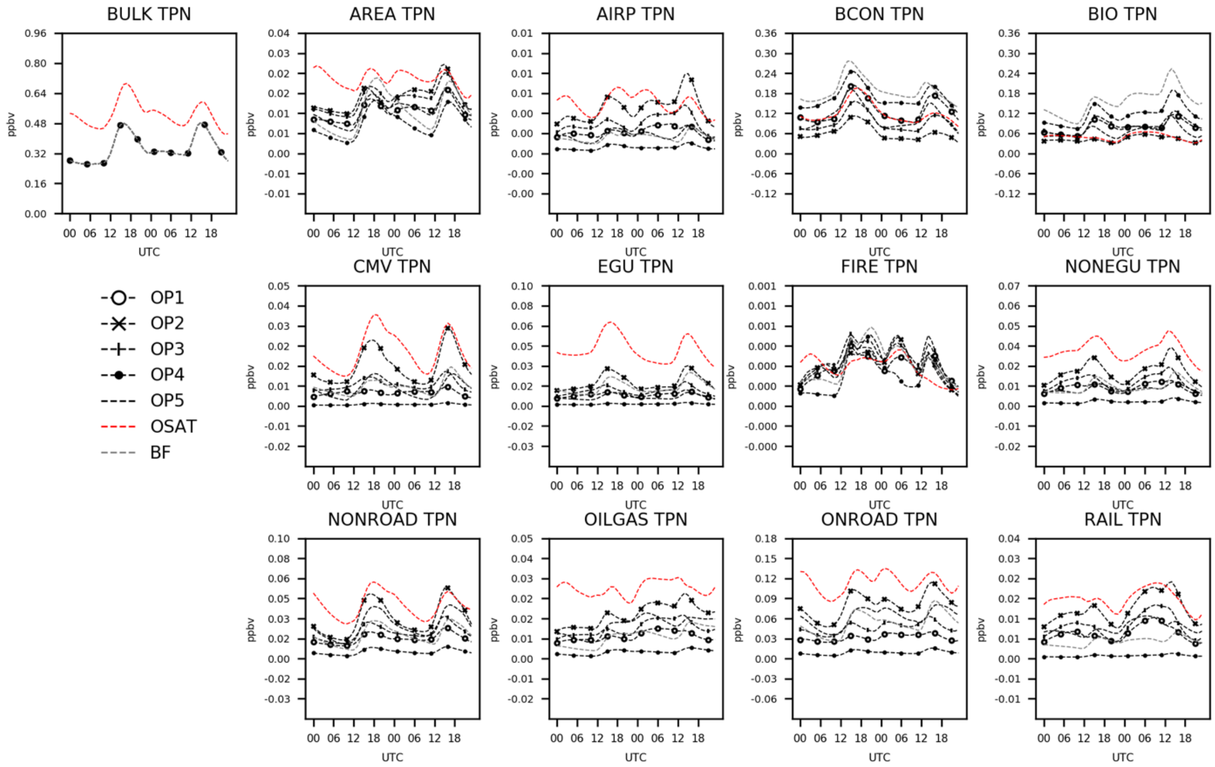

Fig. S3(c) Comparisons of hourly variations of TPN concentrations among seven source apportionment simulations (OP1 to OP5, OSAT, CMAQ-BF) for bulk mixing ratios and selected sector contributions.

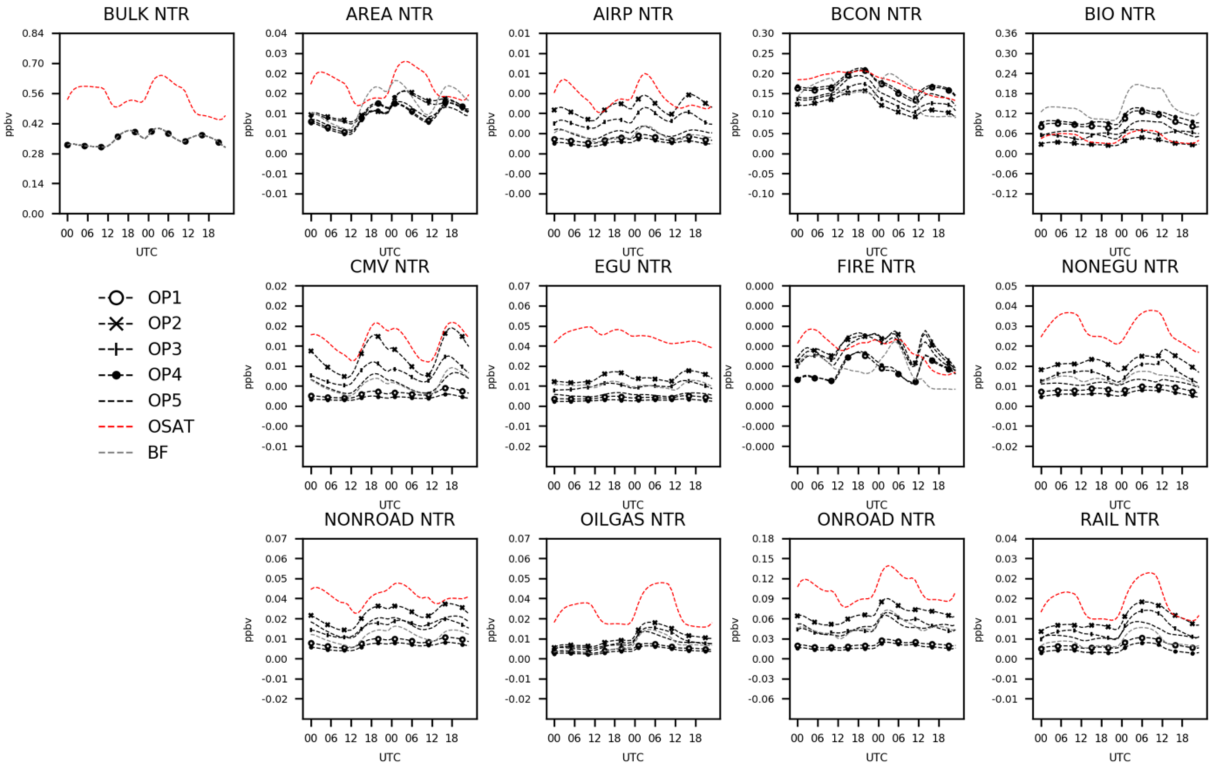

Fig. S3(d) Comparisons of hourly variations of NTR concentrations among seven source apportionment simulations (OP1 to OP5, OSAT, CMAQ-BF) for bulk mixing ratios and selected sector contributions.

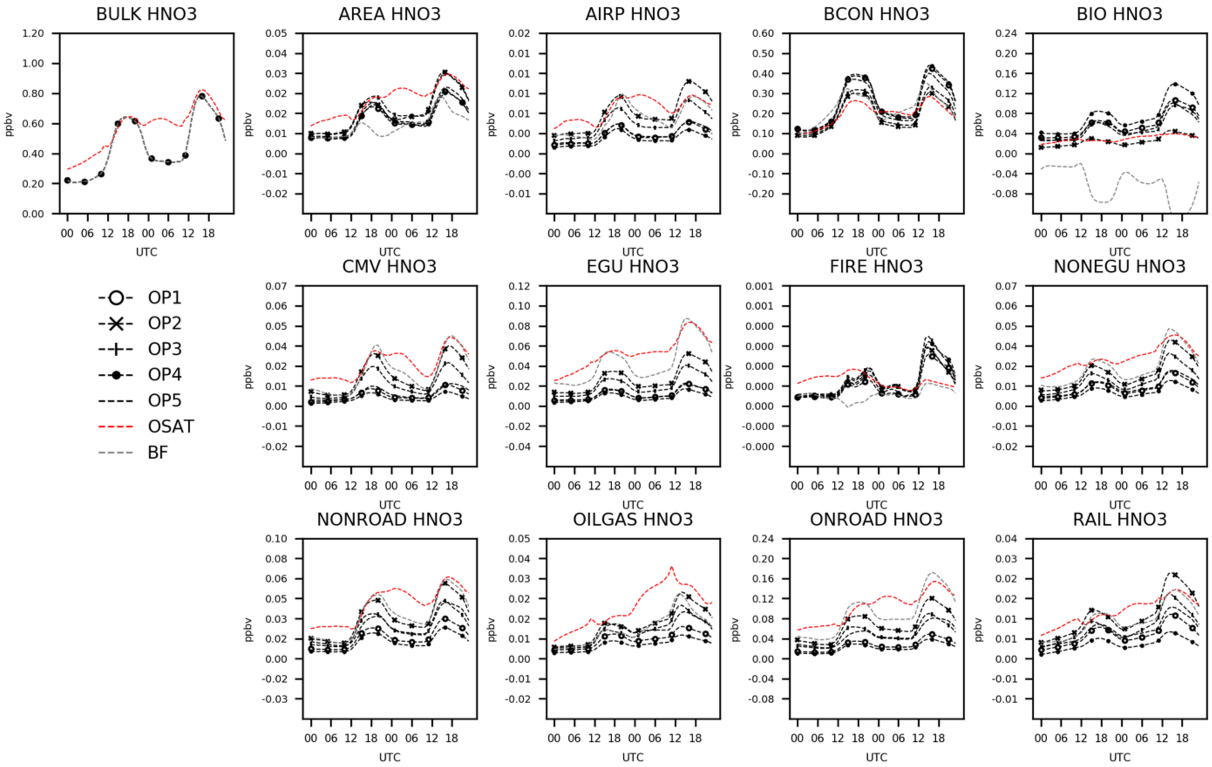

Fig. S3(e) Comparisons of hourly variations of HNO_3 concentrations among seven source apportionment simulations (OP1 to OP5, OSAT, CMAQ-BF) for bulk mixing ratios and selected sector contributions.

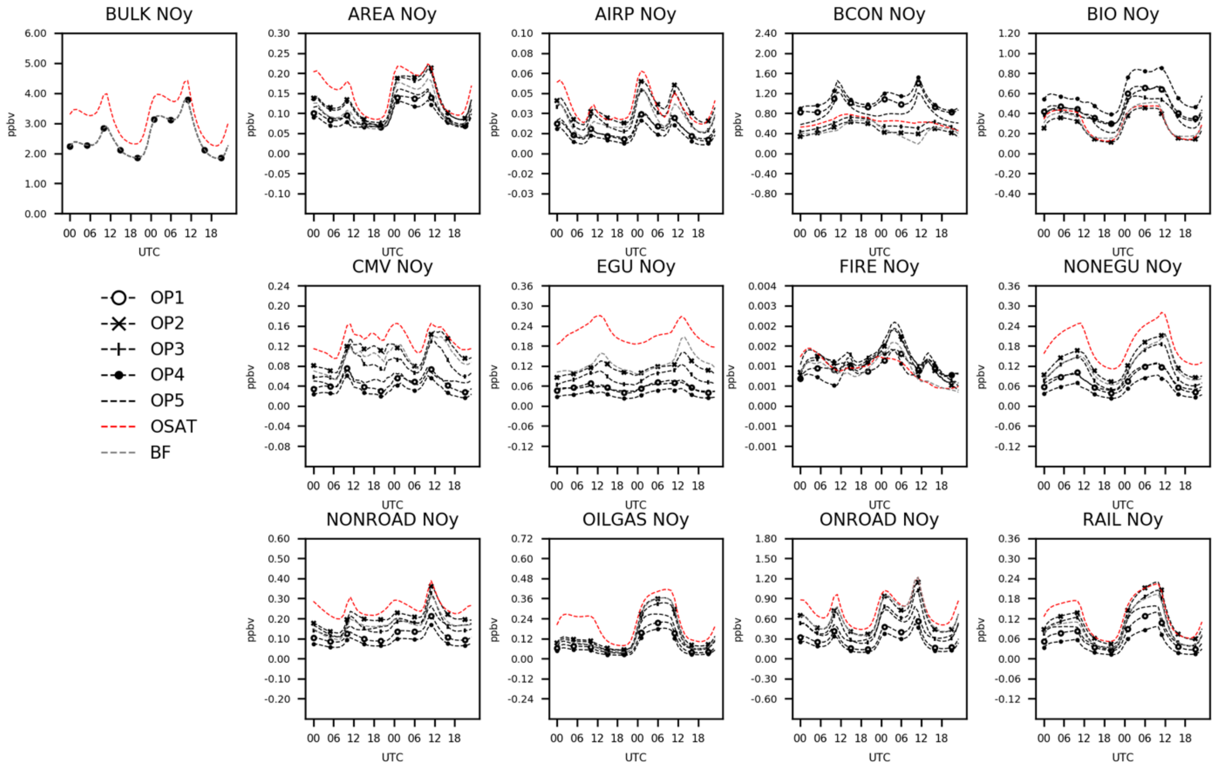

Fig. S3(f) Comparisons of hourly variations of NO_y concentrations among seven source apportionment simulations (OP1 to OP5, OSAT, CMAQ-BF) for bulk mixing ratios and selected sector contributions.

S4. Spatial distribution of source apportionment simulations for monthly averaged MDA8 O_3, RNO_x, and VOCs.

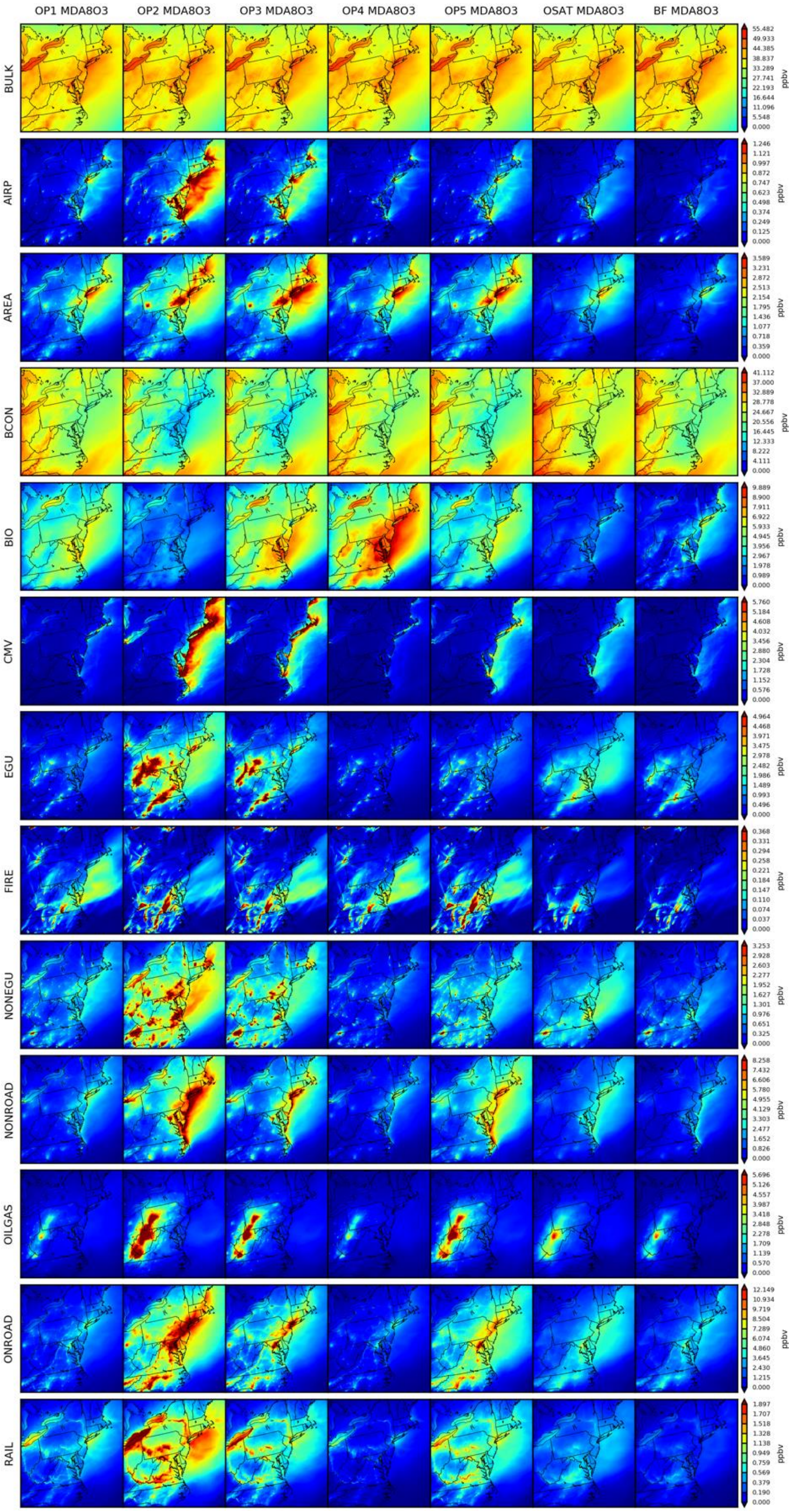

Fig. S4(a) Spatial comparisons of seven simulations for monthly averaged MDA8 O_3 (07/29-08/30).

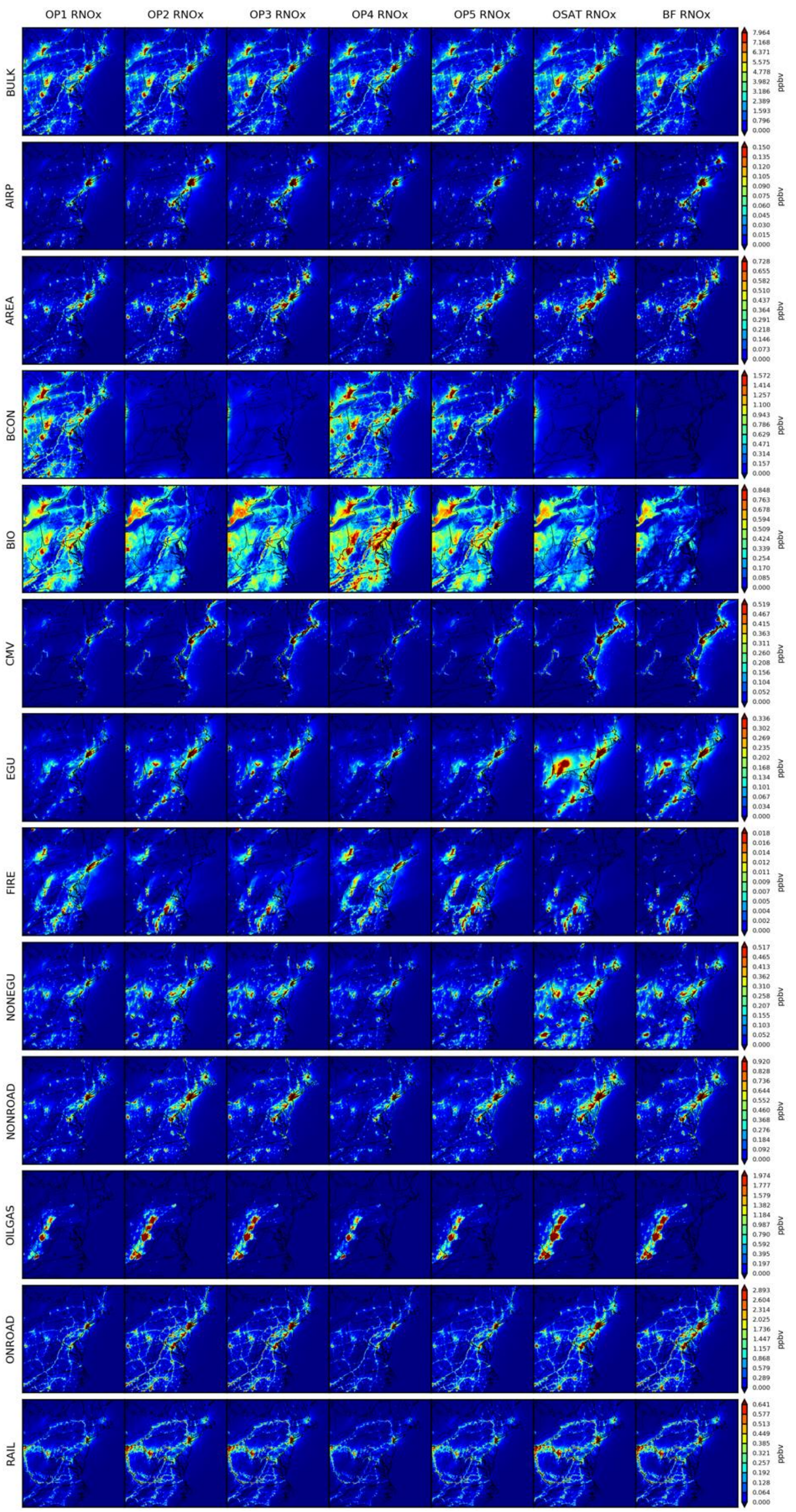

Fig. S4(b) Spatial comparisons of seven simulations for monthly averaged RNO_x (07/29-08/30).

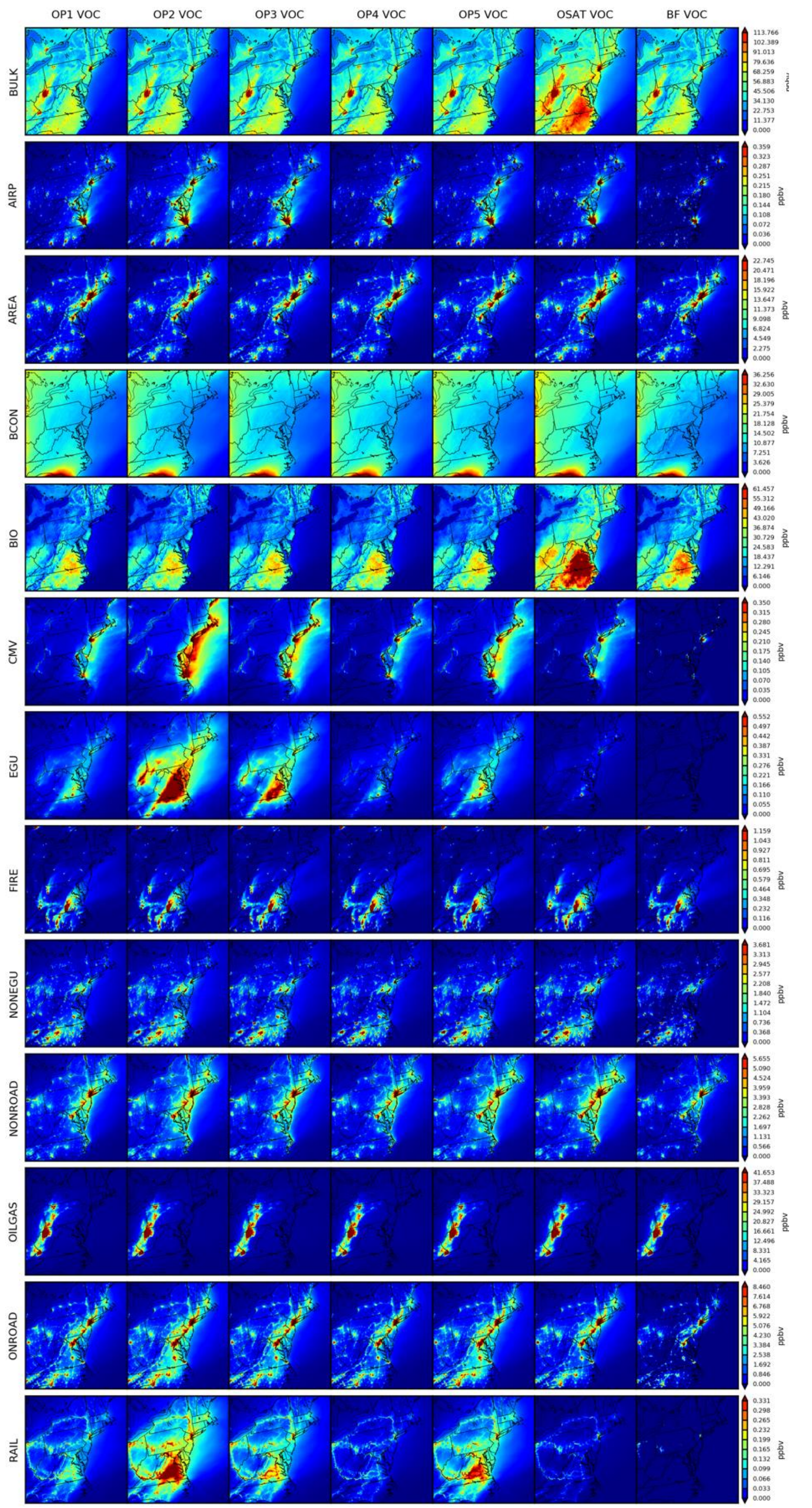

Fig. S4(c) Spatial comparisons of seven simulations for monthly averaged VOCs (07/29-08/30).

S5. Spatial distribution of source apportionment simulations for additional two-day averaged tracked species in Table 4.

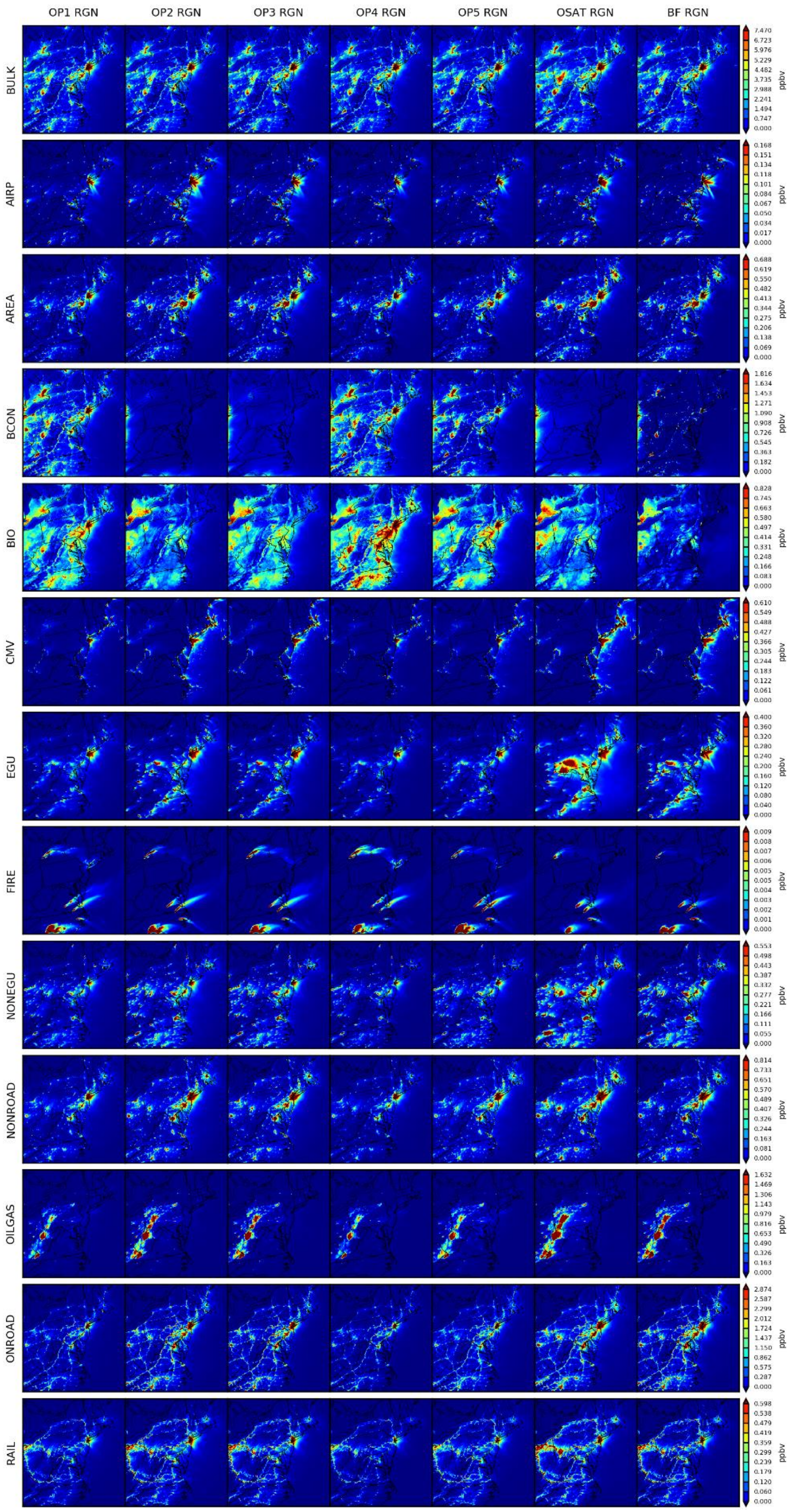

Fig. S5(a) Spatial comparisons of seven simulations for two-day averaged RGN (08/09 and 08/10).

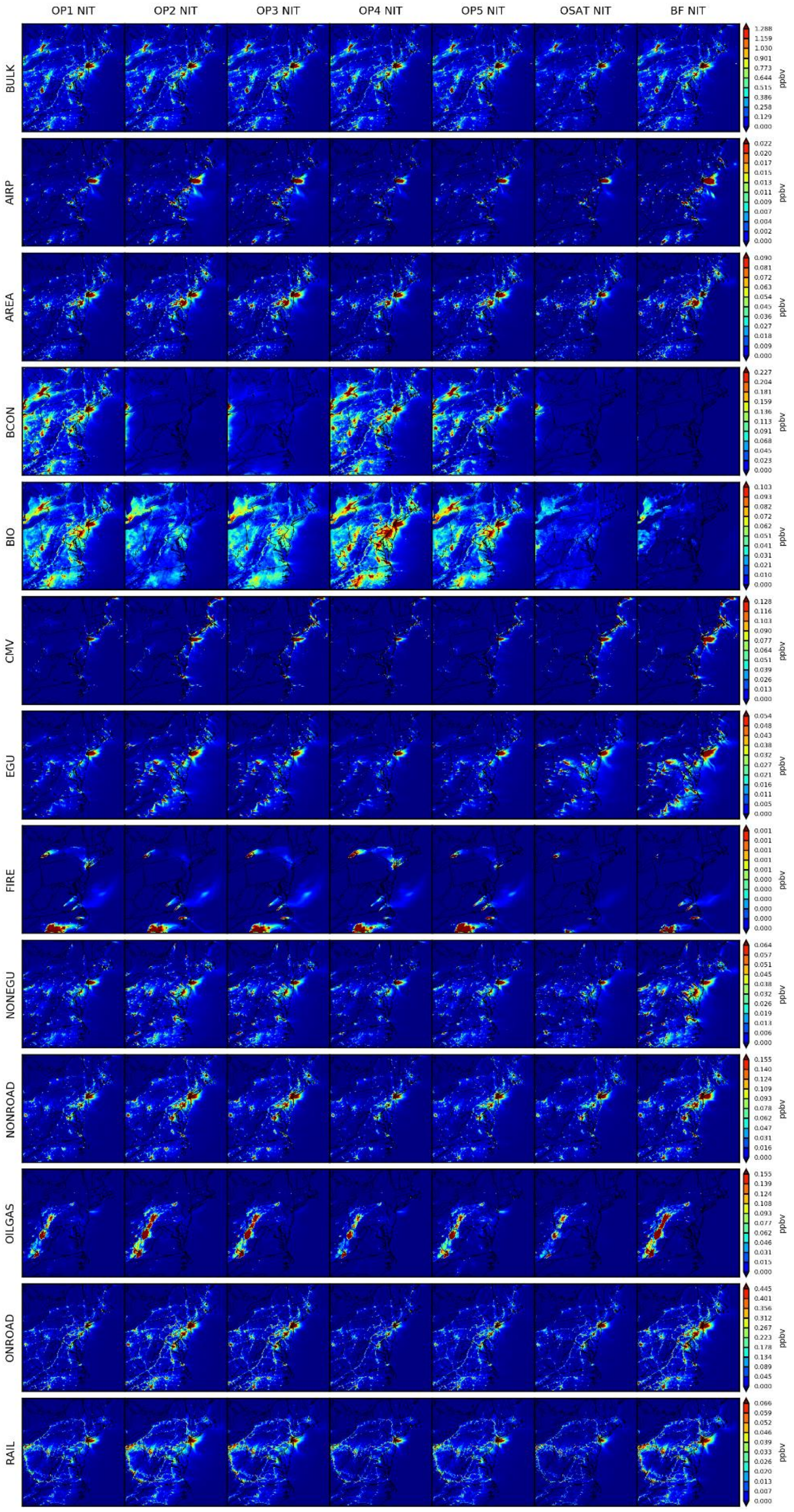

Fig. S5(b) Spatial comparisons of seven simulations for two-day averaged NIT (08/09 and 08/10).

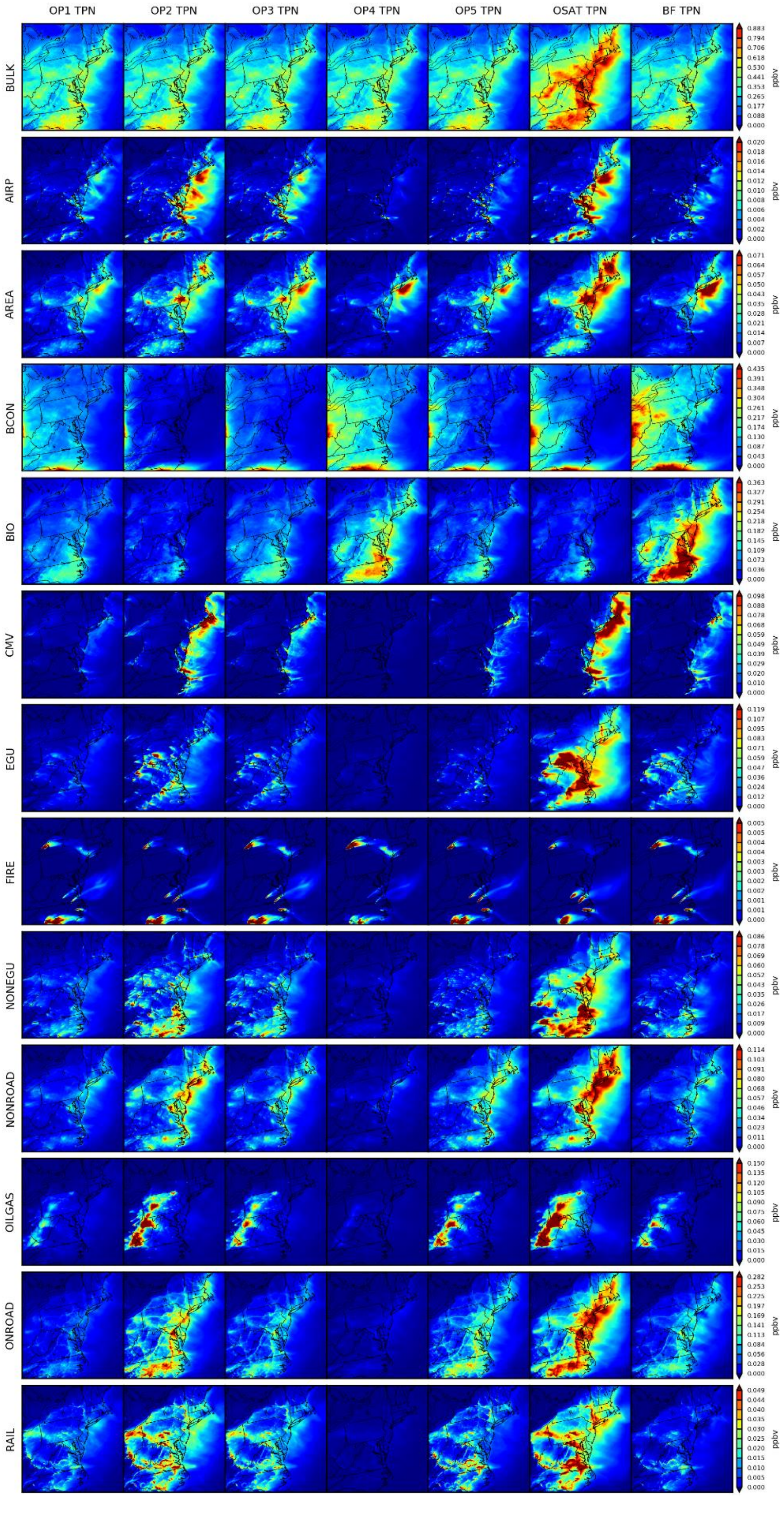

Fig. S5(c) Spatial comparisons of seven simulations for two-day averaged TPN (08/09 and 08/10).

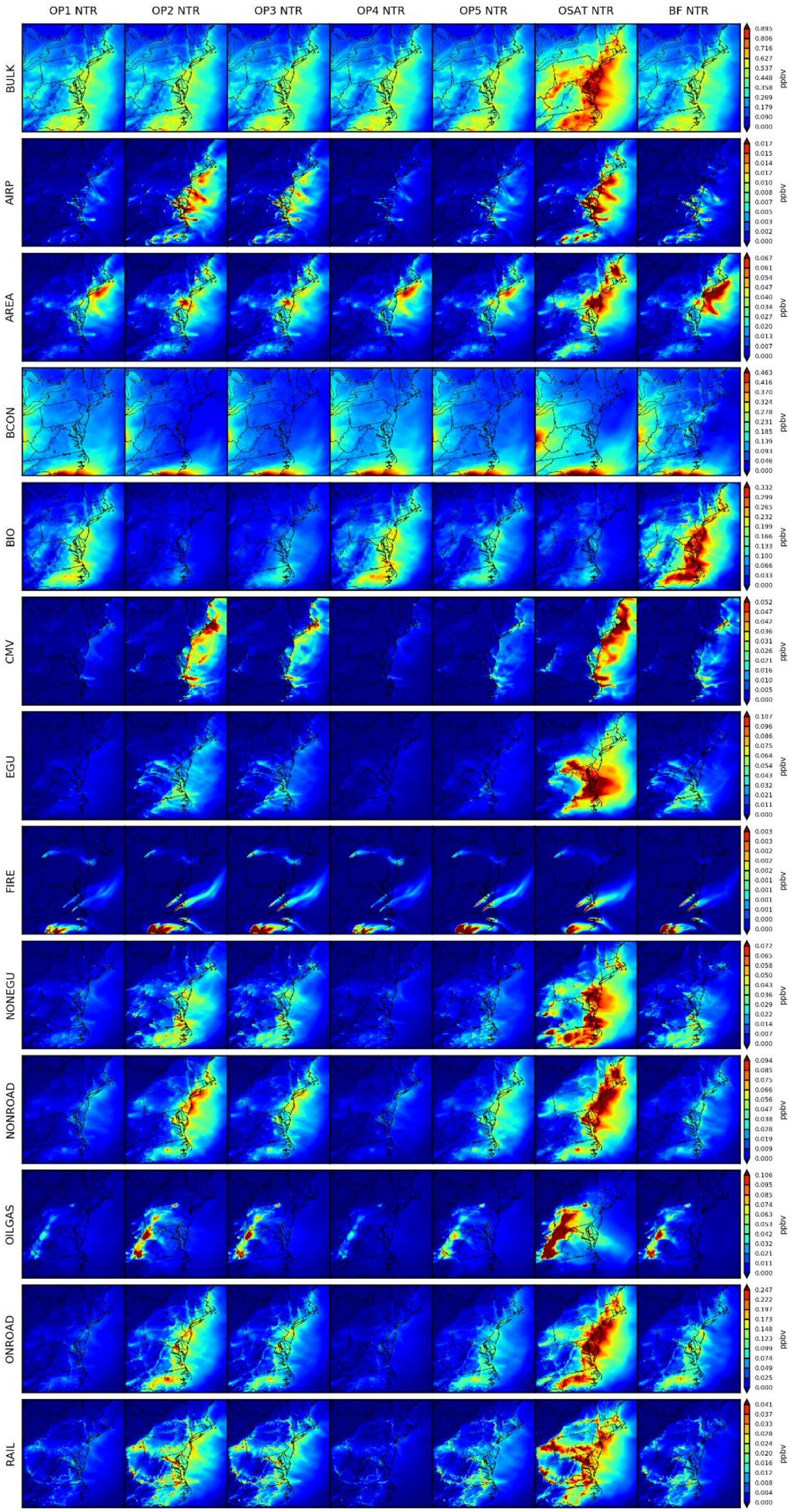

Fig. S5(d) Spatial comparisons of seven simulations for two-day averaged NTR (08/09 and 08/10).

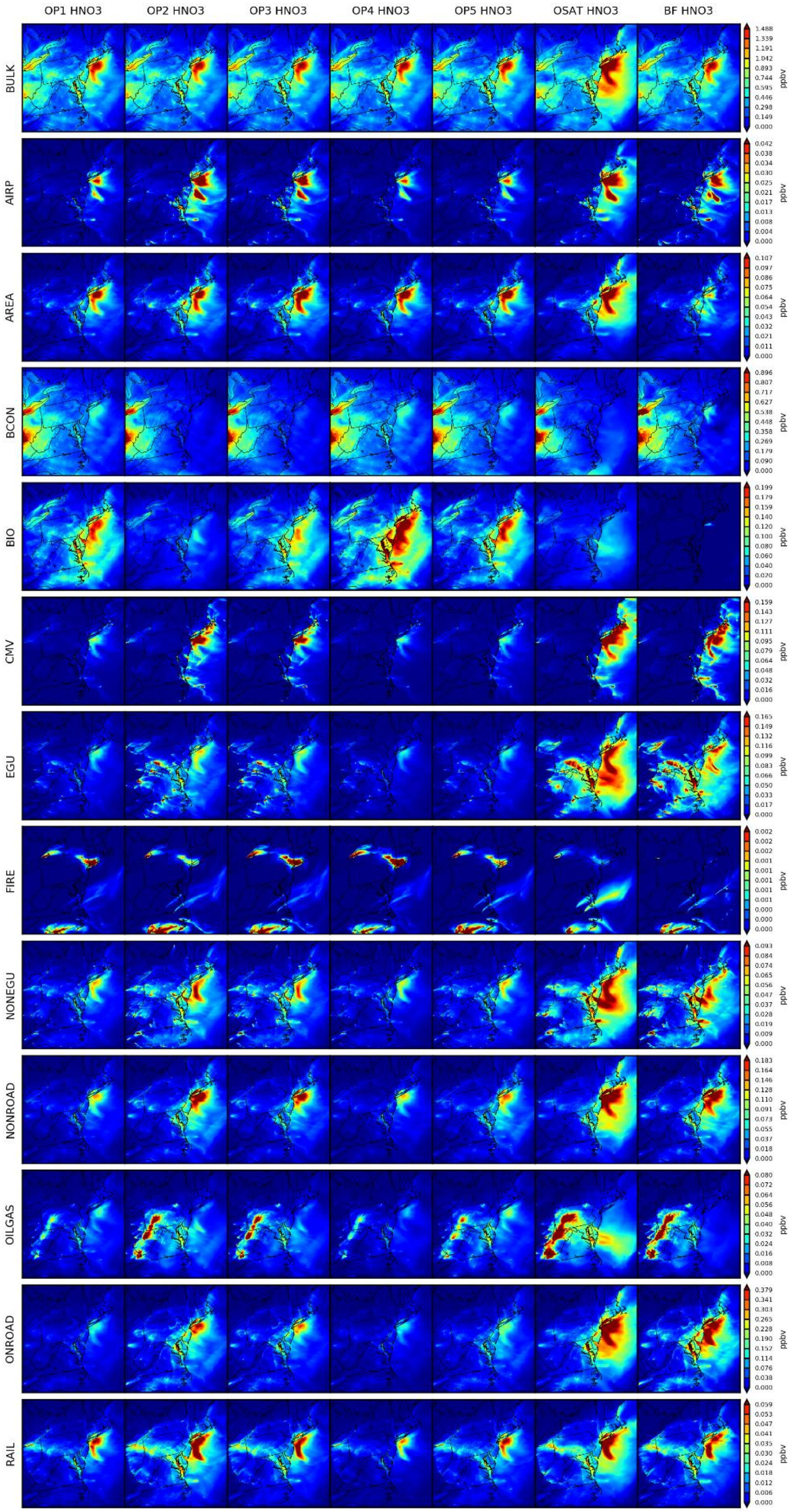

Fig. S5(e) Spatial comparisons of seven simulations for two-day averaged HNO_3 (08/09 and 08/10).

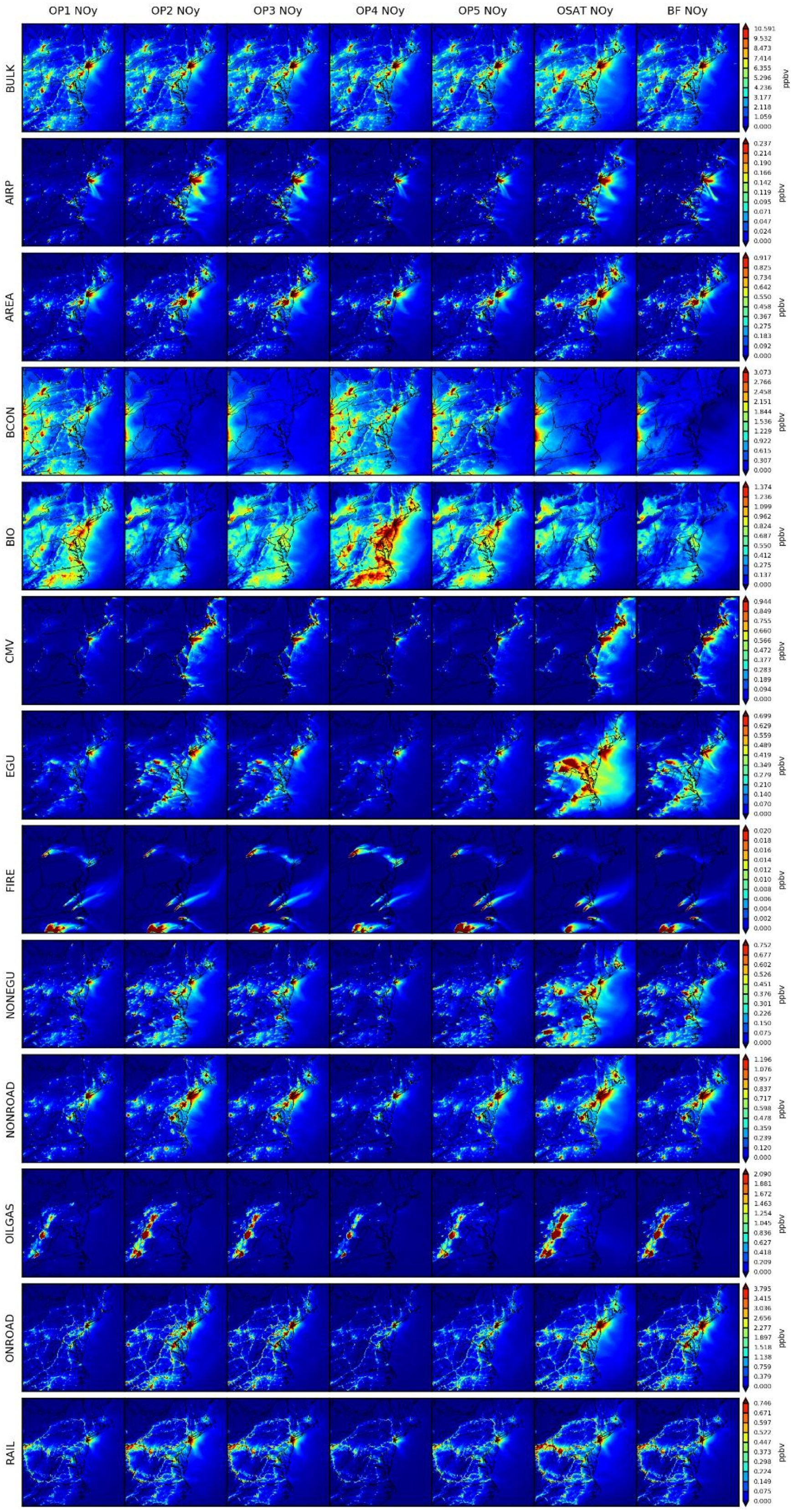

Fig. S5(f) Spatial comparisons of seven simulations for two-day averaged NO_y (08/09 and 08/10).

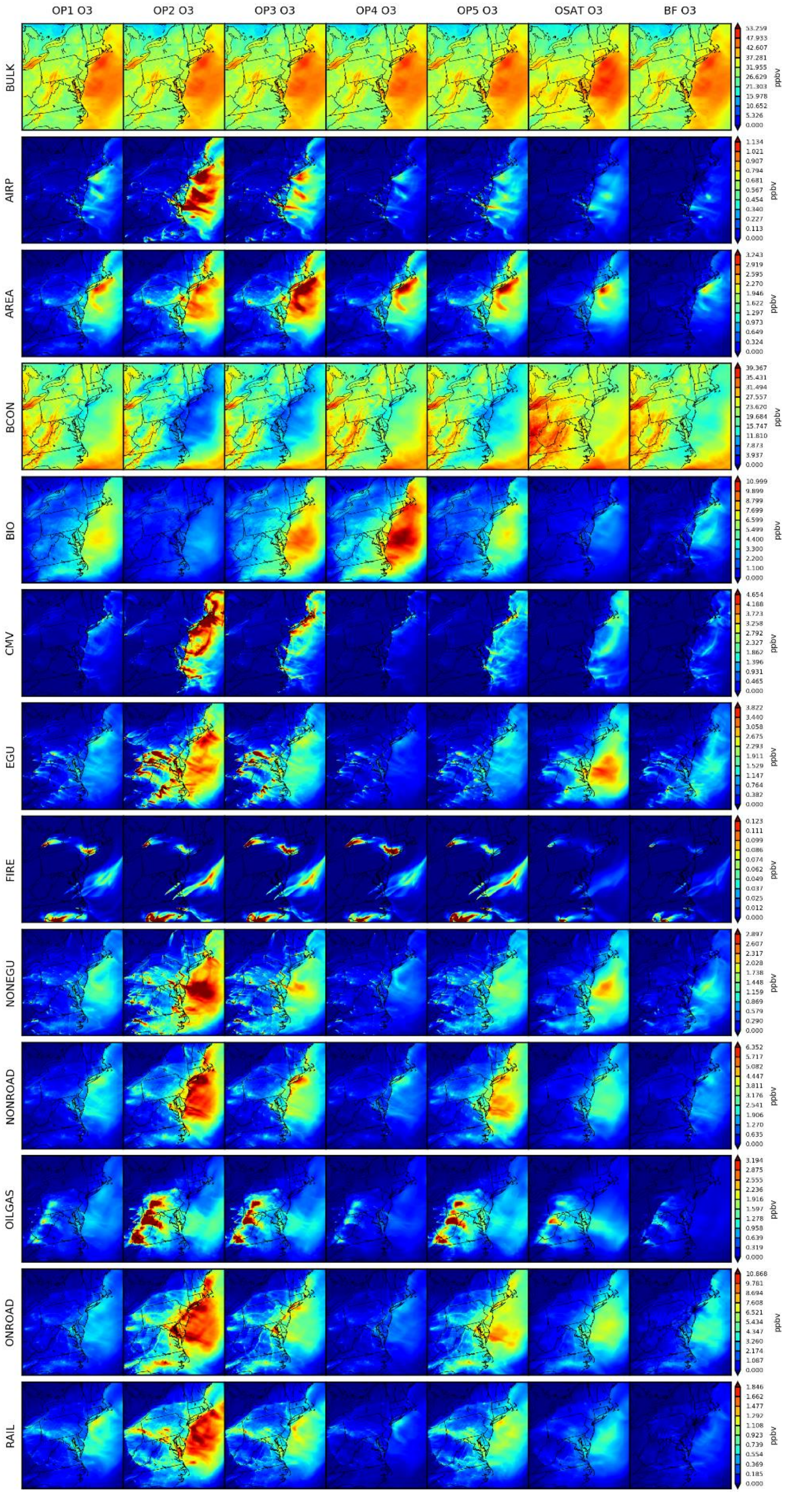

Fig. S5(g) Spatial comparisons of seven simulations for two-day averaged O_3 (08/09 and 08/10).

S6. Temporal and spatial averaged source contributions

Relative contributions could eliminate the dependence of source apportionment methods on their parent models, allowing for insightful comparisons between OSAT, ISAM, and CMAQ-BF. To reduce the nighttime O_3 discrepancy between OSAT and ISAM, the following comparisons employ MDA8 O_3. Figure S6 shows two-day averaged source percentage contributions to (a) MDA8 O_3, (b) RNO_x and (c) VOC for each sector across the domain from sven source apportionment simulations (OP1 to OP5, OSAT and CMAQ-BF). The percent contribution from each sector is calculated as Equation (S1):

$$P_{m,sector} = 100 * \frac{C_{m,sector}}{C_{m,bulk}} \text{ (S1)}$$

Where $P_{m,sector}$ is the percent contribution of each sector for each source apportionment method; $C_{m,sector}$ is the absolute species concentration of each sector; $C_{m,bulk}$ is the bulk species concentration. The detailed percent contributions and absolute concentrations are summarized in Table S2 (a) and (b). In Fig. S6(a), although substantial differences are observed among the different apportionment methods for absolute contributions from each sector, there is closer agreement for yield similar relative proportions of the source contribution. All approaches predict the order of larger sectors consistently, while the order of smaller sectors exhibits differences.

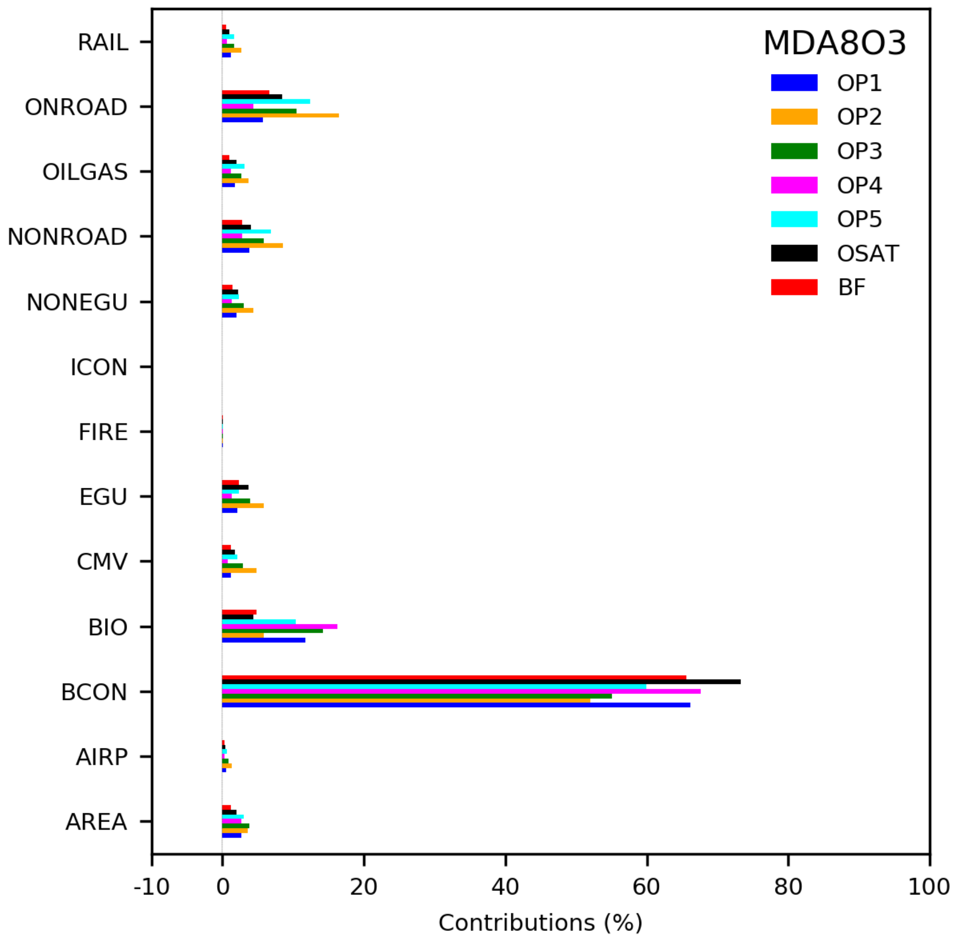

Fig. S6(a) Two-day averaged domain-wide contributions of MDA8 O_3 from each sector for seven source apportionment simulations (OP1 to OP5, OSAT, CMAQ-BF).

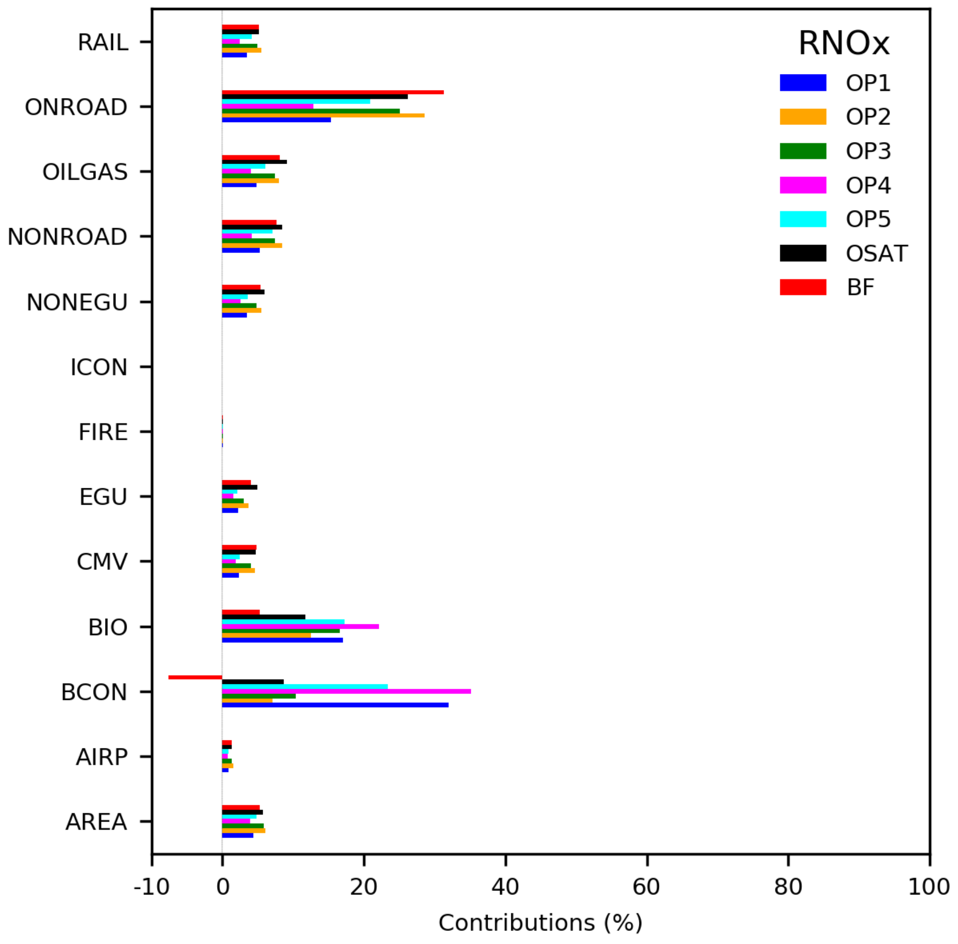

Fig. S6(b) Two-day averaged domain-wide contributions of RNO_x from each sector for seven source apportionment simulations (OP1 to OP5, OSAT, CMAQ-BF).

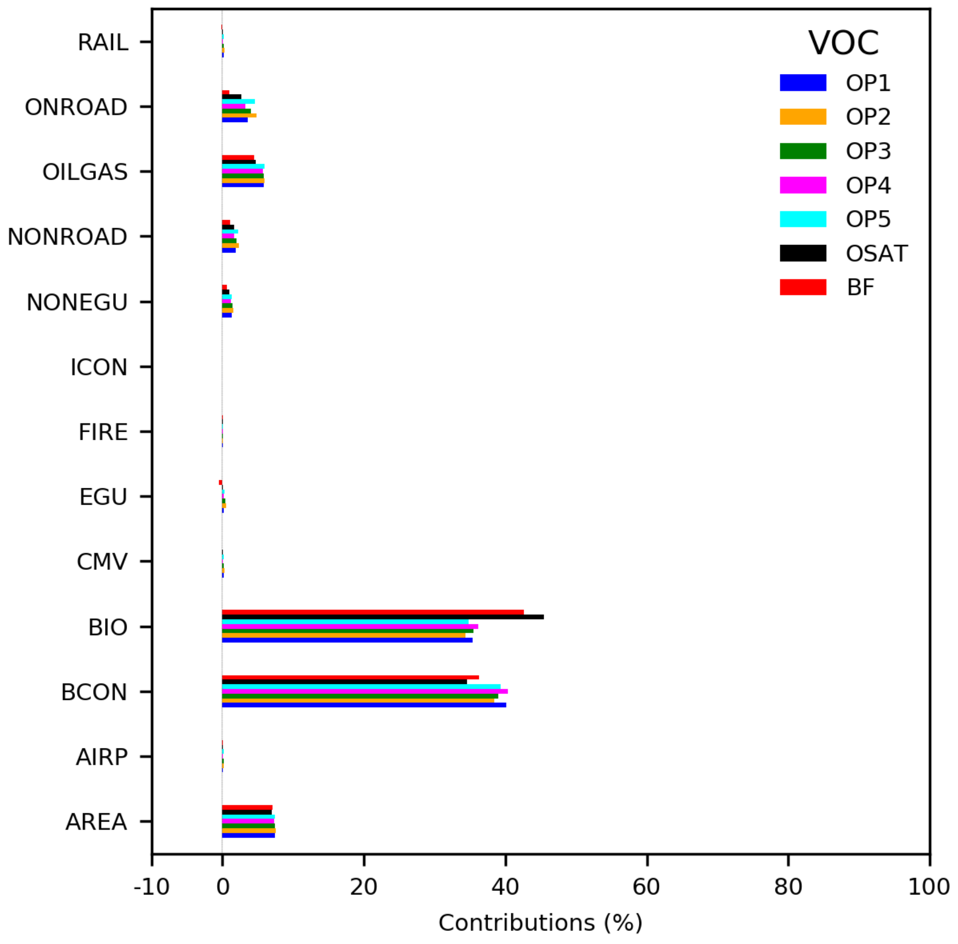

Fig. S6(c) Two-day averaged domain-wide contributions of VOC from each sector for seven source apportionment simulations (OP1 to OP5, OSAT, CMAQ-BF).

Table S2(a). Domain-wide two-day mean percentage contributions (%)

	CASE	AREA	AIRP	BCON	BIO	CMV	EGU	FIRE	ICON	NON EGU	NON ROAD	OIL GAS	ON ROAD	RAIL
MDA8 O3	OP1	2.65	0.48	66.21	11.69	1.25	2.14	0.06	0.00	1.98	3.79	1.72	5.76	1.22
	OP2	3.56	1.31	51.98	5.90	4.79	5.85	0.08	0.00	4.37	8.56	3.66	16.50	2.69
	OP3	3.83	0.84	55.02	14.24	2.90	3.87	0.08	0.00	3.00	5.80	2.71	10.53	1.70
	OP4	2.68	0.34	67.64	16.27	0.78	1.37	0.06	0.00	1.29	2.84	1.19	4.32	0.61
	OP5	3.06	0.59	59.96	10.41	2.14	2.32	0.08	0.00	2.39	6.81	3.08	12.39	1.67
	OSAT	1.96	0.38	73.32	4.32	1.72	3.65	0.03	0.00	2.21	4.05	2.00	8.46	1.01
	BF	1.25	0.26	65.60	4.86	1.16	2.31	0.03	0.00	1.41	2.74	1.00	6.61	0.58
RNOx	OP1	4.34	0.89	31.93	17.07	2.32	2.25	0.04	0.00	3.43	5.31	4.85	15.38	3.44
	OP2	6.02	1.55	7.12	12.50	4.63	3.66	0.05	0.00	5.46	8.45	8.05	28.56	5.54
	OP3	5.87	1.37	10.36	16.56	4.01	3.03	0.05	0.00	4.79	7.39	7.40	25.04	4.91
	OP4	3.93	0.73	35.12	22.13	1.88	1.59	0.04	0.00	2.60	4.20	4.06	12.85	2.50
	OP5	4.82	0.90	23.40	17.25	2.48	2.15	0.05	0.00	3.59	7.05	6.12	20.89	4.19
	OSAT	5.73	1.34	8.66	11.68	4.66	4.97	0.03	0.00	5.99	8.41	9.13	26.25	5.13
	BF	5.23	1.37	-7.65	5.29	4.77	4.07	0.04	-0.08	5.43	7.70	8.08	31.29	5.19
VOC	OP1	7.41	0.12	40.19	35.40	0.13	0.22	0.08	0.00	1.28	1.85	5.79	3.54	0.14
	OP2	7.51	0.17	38.49	34.38	0.25	0.58	0.09	0.00	1.54	2.31	6.00	4.88	0.29
	OP3	7.48	0.14	39.04	35.48	0.17	0.37	0.08	0.00	1.38	2.02	5.88	4.06	0.20
	OP4	7.37	0.11	40.39	36.12	0.10	0.13	0.08	0.00	1.19	1.71	5.72	3.28	0.08
	OP5	7.46	0.13	39.31	34.80	0.16	0.27	0.09	0.00	1.36	2.20	5.94	4.55	0.23
	OSAT	7.02	0.09	34.54	45.43	0.08	0.05	0.06	0.00	1.03	1.62	4.73	2.69	0.03
	BF	7.09	0.04	36.30	42.62	-0.07	-0.52	0.08	-0.01	0.62	1.09	4.45	0.99	-0.19

Table S2(b). Domain-wide two-day mean absolute concentration contributions (ppb)

	CASE	AREA	AIRP	BCON	BIO	CMV	EGU	FIRE	ICON	NON EGU	NON ROAD	OIL GAS	ON ROAD	RAIL
MDA8 O3	OP1	1.25	0.23	31.18	5.51	0.59	1.01	0.03	0.00	0.93	1.78	0.81	2.71	0.57
	OP2	1.68	0.62	24.48	2.78	2.26	2.76	0.04	0.00	2.06	4.03	1.73	7.77	1.26
	OP3	1.80	0.40	25.91	6.71	1.37	1.82	0.04	0.00	1.41	2.73	1.28	4.96	0.80
	OP4	1.26	0.16	31.86	7.66	0.37	0.64	0.03	0.00	0.61	1.34	0.56	2.04	0.29
	OP5	1.44	0.28	28.24	4.90	1.01	1.09	0.04	0.00	1.12	3.21	1.45	5.84	0.78
	OSAT	0.93	0.18	34.91	2.06	0.82	1.74	0.01	0.00	1.05	1.93	0.95	4.03	0.48
	BF	0.59	0.12	30.89	2.29	0.54	1.09	0.01	0.00	0.66	1.29	0.47	3.11	0.27
RNOx	OP1	0.06	0.01	0.43	0.23	0.03	0.03	0.00	0.00	0.05	0.07	0.07	0.21	0.05
	OP2	0.08	0.02	0.10	0.17	0.06	0.05	0.00	0.00	0.07	0.12	0.11	0.39	0.08
	OP3	0.08	0.02	0.14	0.23	0.05	0.04	0.00	0.00	0.07	0.10	0.10	0.34	0.07
	OP4	0.05	0.01	0.48	0.30	0.03	0.02	0.00	0.00	0.04	0.06	0.06	0.18	0.03
	OP5	0.07	0.01	0.32	0.23	0.03	0.03	0.00	0.00	0.05	0.10	0.08	0.28	0.06
	OSAT	0.09	0.02	0.13	0.18	0.07	0.08	0.00	0.00	0.09	0.13	0.14	0.41	0.08
	BF	0.07	0.02	-0.10	0.07	0.07	0.06	0.00	0.00	0.07	0.10	0.11	0.43	0.07
VOC	OP1	3.40	0.06	18.45	16.25	0.06	0.10	0.04	0.00	0.59	0.85	2.66	1.63	0.06
	OP2	3.45	0.08	17.67	15.78	0.12	0.26	0.04	0.00	0.71	1.06	2.76	2.24	0.14
	OP3	3.43	0.06	17.92	16.29	0.08	0.17	0.04	0.00	0.64	0.93	2.70	1.87	0.09
	OP4	3.38	0.05	18.54	16.58	0.04	0.06	0.04	0.00	0.55	0.79	2.63	1.51	0.03
	OP5	3.42	0.06	18.05	15.97	0.08	0.12	0.04	0.00	0.62	1.01	2.73	2.09	0.10
	OSAT	4.37	0.06	21.52	28.30	0.05	0.03	0.04	0.00	0.64	1.01	2.94	1.68	0.02
	BF	3.25	0.02	16.66	19.57	-0.03	-0.24	0.04	-0.01	0.28	0.50	2.04	0.46	-0.09
